# Phase formation capability and compositional design of β-phase multiple rare-earth principal component disilicates

**DOI:** 10.1038/s41467-023-36947-6

**Published:** 2023-03-08

**Authors:** Yixiu Luo, Luchao Sun, Jiemin Wang, Tiefeng Du, Cui Zhou, Jie Zhang, Jingyang Wang

**Affiliations:** 1 Shenyang National Laboratory for Materials Science, Institute of Metal Research, Chinese Academy of Sciences, Shenyang, 110016 China; 2 School of Materials Science and Engineering, University of Science and Technology of China, Shenyang, 110016 China

**Keywords:** Ceramics, Aerospace engineering

## Abstract

A key strategy to design environmental barrier coatings focuses on doping multiple rare-earth principal components into β-type rare-earth disilicates (RE_2_Si_2_O_7_) to achieve versatile property optimization. However, controlling the phase formation capability of (*n*RE_*xi*_)_2_Si_2_O_7_ remains a crucial challenge, due to the complex polymorphic phase competitions and evolutions led by different RE^3+^ combination. Herein, by fabricating twenty-one model (RE^I^_0.25_RE^II^_0.25_RE^III^_0.25_RE^IV^_0.25_)_2_Si_2_O_7_ compounds, we find that their formation capability can be evaluated by the ability to accommodate configurational randomness of multiple RE^3+^ cations in β-type lattice while preventing the β-to-γ polymorphic transformation. The phase formation and stabilization are controlled by the average RE^3+^ radius and the deviations of different RE^3+^ combinations. Subsequently, based on high-throughput density-functional-theory calculations, we propose that the configurational entropy of mixing is a reliable descriptor to predict the phase formation of β-type (*n*RE_*xi*_)_2_Si_2_O_7_. The results may accelerate the design of (*n*RE_*xi*_)_2_Si_2_O_7_ materials with tailored compositions and controlled polymorphic phases.

## Abstract (web summary)

Developing high-entropy environmental barrier coatings is challenged by the intricacy to form stable multicomponent materials. Here the authors report the phases of high-entropy β-RE_2_Si_2_O_7_ could be precisely designed by monitoring the entropy descriptor and the cationic radii on rare earth sites.

## Introduction

Multifunctional thermal and environmental barrier coatings (TEBC) are attracting much attention in the gas turbine technology, which are expected to protect the turbine components made of SiC_f_/SiC ceramic matrix composites (CMCs) from environmental degradation and thermal attack in the high-temperature water-vapor-rich combustion environment, and thus allow for higher inlet temperature, higher core-power, and higher fuel efficiency of turbine engines^1^. Rare-earth disilicates are among the most promising candidates that have been considered and tested for the TEBC applications^2,3^. For instance, a highly potential TEBC system is conceptualized in multilayer architectures, wherein the rare-earth disilicates (RE_2_Si_2_O_7_) having matched coefficient of thermal expansion (CTE = (4–5) × 10^−6^ K^−1^)^4,5^ with that of SiC_f_/SiC CMCs substrates (CTE = (4.5–5.5) × 10^−6^ K^−1^)^6^ are necessarily used as an intermediate layer to buffer the CTE gap between the substrates and the top coats (typically, rare-earth monosilicates RE_2_SiO_5_ with CTE = (6–10) × 10^−6^ K^−1^)^7^, for the purpose of minimizing the inter-layer thermal stress and thus prevent coating delamination during thermal cycling^8^. Nevertheless, most RE_2_Si_2_O_7_ disilicates undergo complex phase transformations (α, β, γ, δ, A, F and G) with the variation of temperatures^9^, wherein the associated volume change or the CTE mismatch of the product phases will cause catastrophic cleaving or delamination of the coatings; except for RE_2_Si_2_O_7_ (RE = Yb, Lu and Sc), which exhibit good stability of β-type polymorph up to their melting points. Consequently, the list of conventional single-RE-component RE_2_Si_2_O_7_ ceramics suitable for TEBC applications are narrowed down to β-type RE_2_Si_2_O_7_ (RE = Yb, Lu and Sc) compounds, and some co-doped solid solutions containing these RE elements^1^; yet previous research has shown that their performance under aggressively coupled attacks from hot steam, molten CMAS and thermal stress cannot meet the requirements of advanced TEBC systems^10^. Therefore, RE_2_Si_2_O_7_ materials with optimized overall performance, including good phase stability, are in high demands.

Very recently, the concept of multicomponent (or high-entropy) modification of RE_2_Si_2_O_7_ candidates brings new opportunities. It enables the search and property-optimization of single-phase multi-RE-principal-component RE_2_Si_2_O_7_ (thereafter denoted as (*n*RE_*xi*_)_2_Si_2_O_7_) with high compositional disorder over a massively expanded and unexplored compositional space, beyond the already-known single-RE-principal-component RE_2_Si_2_O_7_ compounds. The concept of multicomponent materials was originally proposed in metallic alloys by Yeh et al. in 2004^11^, then extended into ceramics by Rost et al. in 2015^12^, and has since penetrated to include borides^13^, carbides^14–18^, nitrides^19^, sulfides^20^, silicides^21^, and other complex oxides^22–27^. Multicomponent systems tend to have interesting qualities, including high mixing entropy, sluggish diffuson kinetics, severe lattice distortions and the cocktail effect^28^; which lead to remarkable properties, such as high melting points, superior phase stability, high hardness, amorphous-like thermal conductivity, and excellent wear and corrosion resistance, etc.^29^. For RE_2_Si_2_O_7_ materials, previous explorations have shown that some rare-earth elements act as the key RE ingredients to improve specific properties, such as the element Ho to the enhanced water-vapor resistance, and Lu to the enhanced CMAS resistance, just to name a few^30^. Therefore, a promising outcome, which is also the prime goal, of designing (*n*RE_*xi*_)_2_Si_2_O_7_ materials lie in the anticipation of synergistic optimization on the structural stability, mechanical, thermal and corrosion-resistant properties, by mix-and-matching the key RE ingredients; and the overall performance of the (*n*RE_*xi*_)_2_Si_2_O_7_ materials can further be fine-tuned by elaborately tailoring the compositions. To date, several (*n*RE_*xi*_)_2_Si_2_O_7_ solid solutions have been synthesized and examined to show improved high-temperature thermal stability, lower thermal conductivity, excellent CTE match with SiC_f_/SiC CMC, and enhanced resistance to water-vapor corrosion or CMAS attack, which outperform or at least on a par with the conventional single-RE-principal-component RE_2_Si_2_O_7_ counterparts for TEBC applications^31–33^.

A crucial challenge for the design of (*n*RE_*xi*_)_2_Si_2_O_7_ materials is the intricacy to pinpoint whether a given composition could be synthesized into a single phase having a specifically controlled structure, i.e., hopefully the β-type structure showing good high-temperature phase stability and matched CTE with SiC_f_/SiC CMC. Hitherto, the design of β-type (*n*RE_*xi*_)_2_Si_2_O_7_ compounds has been largely led by the rule-of-thumb manner, whereas the fundamental mechanisms governing their phase formation capability remains unveiled. The obstacle mainly comes from the versatile combinations of multiple rare-earth elements, as well as the abundant polymorphic types with low energy barriers upon polymorphic transformation, such as the β-to-γ polymorphic transformation. To address this issue, one could find some inspirations from previous works on multicomponent binary metal oxides, carbides, diborides, etc., which have revealed that the formation and stabilization of these materials might be influenced by various factors, such as the differences in atomic radius, molar content, lattice constants, valence electron concentration of the doping sites; or the doping sequence, the number of formulas in a unit cell, the mixing enthalpy, the configurational entropy, etc.^13,25,34–38^. These findings cast light on the phase formation and stabilization of multicomponent materials; however, the understandings are derived from materials with relatively simple crystal structures, and thus cannot be directly applied in the (*n*RE_*xi*_)_2_Si_2_O_7_ materials showing complex crystal structures and various polymorphic types with low transformation barrier. Therefore, it remains an imperative demand to investigate the mechanisms of phase formation for β-type (*n*RE_*xi*_)_2_Si_2_O_7_ materials. Four-RE-principal-component RE_2_Si_2_O_7_ could be used as model materials to represent the (*n*RE_*xi*_)_2_Si_2_O_7_ family, as their configurational entropy of mixing are considered high enough to bring substantially tuned mechanical, thermal and corrosion-resistant properties^39,40^. The mechanisms of phase formation obtained from these model materials can be generalized to guide the compositional design of other (*n*RE_*xi*_)_2_Si_2_O_7_ compounds.

In this study, twenty-one four-RE-principal-component (RE^I^_0.25_RE^II^_0.25_RE^III^_0.25_RE^IV^_0.25_)Si_2_O_7_ (RE = Y, La, Ce, Eu, Gd, Tb, Dy, Ho, Er, Tm, Yb, and Lu) are synthesized, and their phase compositions are examined through X-ray diffraction (XRD) measurements, Rietveld refinement analysis, scanning electron microscope (SEM) observation as well as transmission electron microscope (TEM) characterizations. The phase formation capability is investigated, and its dependencies with respect to the average RE^3+^ radius ($\bar{r}$) and the deviations (*σ*_*r*_) of different RE^3+^ combinations are analyzed. Subsequently, the distribution of mixing energy for four prototype compositions is calculated via high-throughput density functional theory (DFT) calculations, by constructing ensembles that contain several hundreds of representative configurations. The configurational entropy of mixing is calculated, which leads to a descriptor that could describe whether a given composition can form β-type structure with homogeneous RE elemental distributions, and simultaneously captures whether the configurational entropy of mixing has a positive or negative contribution to the β → γ polymorphic transformation. Finally, the strategies to design (*n*RE_*xi*_)_2_Si_2_O_7_ materials with controlled phase formation capability and high-temperature stability are discussed.

## Results

### Phase formation of (RE^I^_0.25_RE^II^_0.25_RE^III^_0.25_RE^IV^_0.25_)_2_Si_2_O_7_

Twenty-one four-RE-principal-component RE_2_Si_2_O_7_ samples are synthesized according to the stoichiometry of (RE^I^_0.25_RE^II^_0.25_RE^III^_0.25_RE^IV^_0.25_)_2_Si_2_O_7_ (RE = Y, La, Ce, Eu, Gd, Tb, Dy, Ho, Er, Tm, Yb, and Lu). As inferred from the phase diagram of single-RE-principal-component RE_2_Si_2_O_7_^9^, Yb^3+^ or Lu^3+^ seem to be necessary ingredients to drive the formation of β-type polymorphic structure. And hence, the different multi-RE^3+^ combinations are designed to include either Yb^3+^ or Lu^3+^, or both; while the other RE^3+^ species, which do not exhibit β-type single-RE-principal-component RE_2_Si_2_O_7_ per se, are sampled across the lanthanides and yttrium. In this way, we might be able to pinpoint the extent to which the randomness/disorder brought by the “alien” RE elements can be tolerated in the β-type lattice, while maintaining systematic discrepancy in the average RE^3+^ cationic radius as well as their deviations. The rationale of designing these compositions is extensively explained in the Supplementary Note 1.

**Fig. 1:**
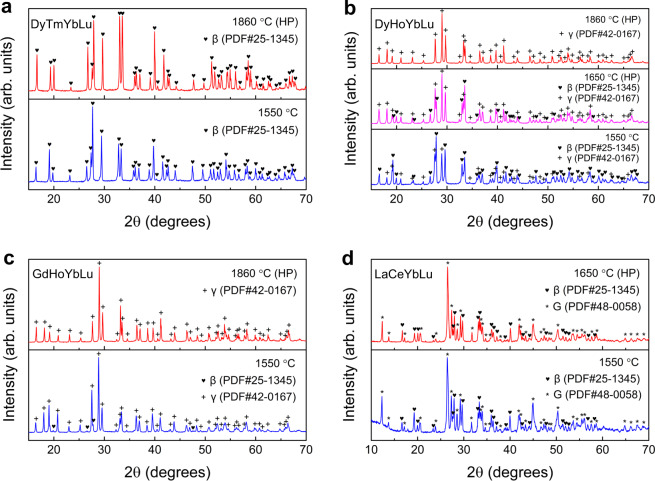
XRD patterns of the synthesized samples. Results for the composition of **a** (Dy_0.25_Tm_0.25_Yb_0.25_Lu_0.25_)_2_Si_2_O_7_, **b** (Dy_0.25_Ho_0.25_Yb_0.25_Lu_0.25_)_2_Si_2_O_7_, **c** (Gd_0.25_Ho_0.25_Yb_0.25_Lu_0.25_)_2_Si_2_O_7_, and **d** (La_*x*1_Ce_*x*2_Yb_*x*3_Lu_*x*4_)_2_Si_2_O_7_ (*x*1 + *x*2 + *x*3 + *x*4 = 1) samples prepared at 1550, 1650, or 1860 °C. “HP” denotes the XRD patterns for bulk samples fabricated via hot-pressing sintering method.

The XRD patterns for all the prepared samples are presented in Fig. 1 and Supplementary Figs. 1–4, which are collected after phase formation at relatively lower temperature (i.e., 1550 °C) followed by sufficient polymorphic transformation at elevated temperature (i.e., above 1800 °C). SEM energy dispersive spectrometer (EDS) point analysis are performed on the hot-pressed bulk samples to quantitatively illustrate the distribution of RE elements, and results are presented in Table 1. Interestingly, the twenty-one compositions could be divided into three categories, based on their finally stabilized polymorphic types at the highest synthesis temperature: (I) single-phase β-type (RE^I^_0.25_RE^II^_0.25_RE^III^_0.25_RE^IV^_0.25_)_2_Si_2_O_7_ solid solution (Fig. 1a and Supplementary Fig. 1); (II) single-phase γ-type (RE^I^_0.25_RE^II^_0.25_RE^III^_0.25_RE^IV^_0.25_)_2_Si_2_O_7_ solid solution (Fig. 1b, c and Supplementary Figs. 2, 3); (III) the designed composition cannot form any polymorphic type of (RE^I^_0.25_RE^II^_0.25_RE^III^_0.25_RE^IV^_0.25_)_2_Si_2_O_7_ solid solution (Fig. 1d and Supplementary Fig. 4).

**Table 1:** The atomic percentage of RE elements (%) in different samples

Sample	Phase	La	Ce	Gd	Dy	Ho	Tm	Yb	Lu	Ratio of RE elements
DyTmYbLu (1860 °C HP)	β	/	/	/	5.19 ± 0.09	/	5.05 ± 0.22	4.73 ± 0.09	4.68 ± 0.09	Dy_0.26_Tm_0.26_Yb_0.24_Lu_0.24_
DyHoYbLu (1650 °C HP)	β or γ	/	/	/	5.77 ± 0.10	5.49 ± 0.21	/	5.29 ± 0.06	4.74 ± 0.20	Dy_0.27_Ho_0.26_Yb_0.25_Lu_0.22_
DyHoYbLu (1860 °C HP)	γ	/	/	/	5.32 ± 0.11	5.21 ± 0.06	/	4.83 ± 0.07	4.59 ± 0.06	Dy_0.27_Ho_0.26_Yb_0.24_Lu_0.23_
GdHoYbLu (1860 °C HP)	γ	/	/	5.31 ± 0.08	/	4.83 ± 0.05	/	4.92 ± 0.10	4.71 ± 0.09	Gd_0.27_Ho_0.24_Yb_0.25_Lu_0.24_
LaCeYbLu (1650 °C HP)	β	1.04 ± 0.07	1.33 ± 0.07	/	/	/	/	9.60 ± 0.42	10.20 ± 0.27	La_0.05_Ce_0.06_Yb_0.43_Lu_0.46_
LaCeYbLu (1650 °C HP)	G	7.87 ± 0.08	7.98 ± 0.17	/	/	/	/	3.60 ± 0.16	3.12 ± 0.09	La_0.35_Ce_0.35_Yb_0.16_Lu_0.14_

Note: Composition identification in different bulk samples is done based on SEM-EDS point analysis. The brackets give the fabrication condition of the bulk samples, wherein “HP” denotes the hot-pressing sintering method. For instance, “1860 °C HP” means that the bulk sample was fabricated via hot-pressing sintering method at 1860 °C.

As shown in Fig. 1a, all the detected peaks for (Dy_0.25_Tm_0.25_Yb_0.25_Lu_0.25_)_2_Si_2_O_7_ samples could be indexed to β-Yb_2_Si_2_O_7_ (PDF#25-1345). The homogeneous distribution of the RE elements is further confirmed by the SEM-EDS analysis, wherein the atomic ratios of Dy:Tm:Yb:Lu are measured to be nearly 1:1:1:1. These observations illustrate that both the as-synthesized powders and the corresponding hot-pressed bulk material are pure single-phase β-(Dy_0.25_Tm_0.25_Yb_0.25_Lu_0.25_)_2_Si_2_O_7_ solid solutions.

**Fig. 2:**
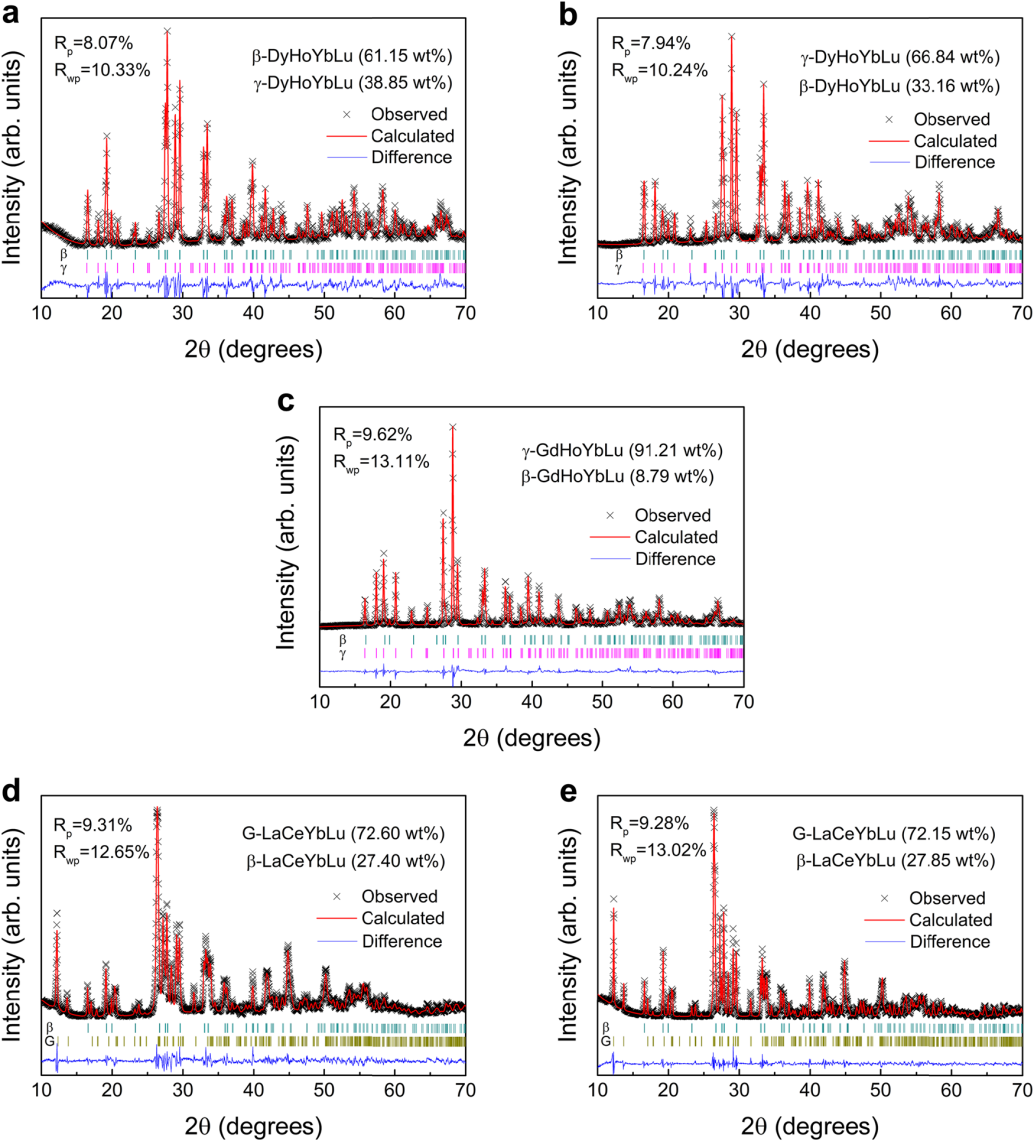
Rietveld refinement of room-temperature XRD patterns for the studied materials. **a** Results for the as-synthesized powders of (Dy_0.25_Ho_0.25_Yb_0.25_Lu_0.25_)_2_Si_2_O_7_ prepared at 1550 °C. **b** Results for the bulk sample of (Dy_0.25_Ho_0.25_Yb_0.25_Lu_0.25_)_2_Si_2_O_7_ fabricated via hot-pressing sintering method at 1650 °C. **c** Results for the as-synthesized powders of (Gd_0.25_Ho_0.25_Yb_0.25_Lu_0.25_)_2_Si_2_O_7_ prepared at 1550 °C. **d** Results for the as-synthesized powders of (La_x1_Ce_x2_Yb_x3_Lu_x4_)_2_Si_2_O_7_ prepared at 1550 °C. **e** Results for the bulk sample of (La_x1_Ce_x2_Yb_x3_Lu_x4_)_2_Si_2_O_7_ fabricated via hot-pressing sintering method at 1650 °C. The reliability factors *R*_p_ and *R*_wp_ are also presented in the figures. The short vertical lines in dark cyan, magenta and dark yellow color at the bottom of each panel denote the positions of XRD peaks of β-RE_2_Si_2_O_7_, γ-RE_2_Si_2_O_7_, and G-RE_2_Si_2_O_7_, respectively.

**Fig. 3:**
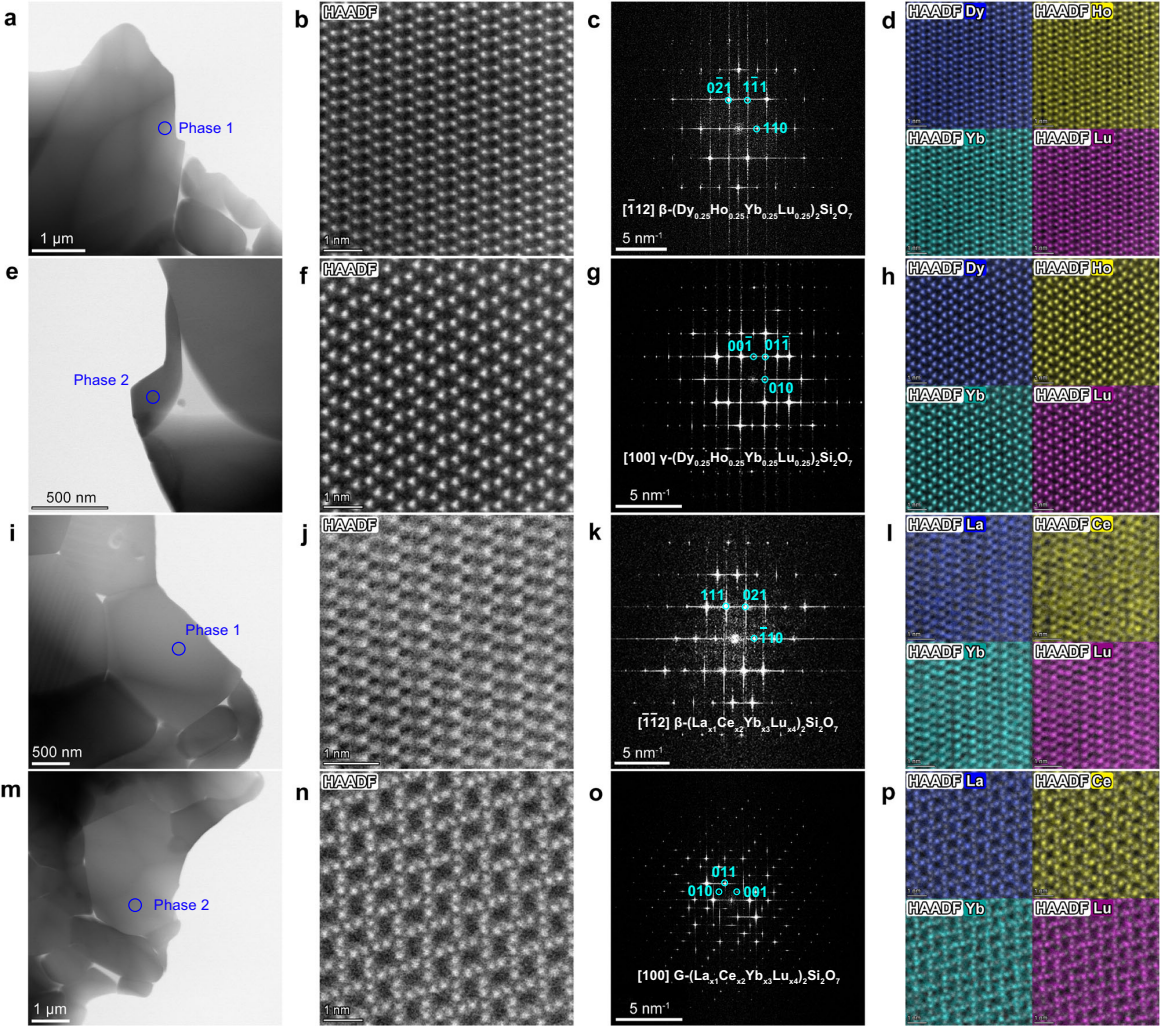
TEM characterization results of (Dy_0.25_Ho_0.25_Yb_0.25_Lu_0.25_)_2_Si_2_O_7_ and (La_x1_Ce_x2_Yb_x3_Lu_x4_)_2_Si_2_O_7_ bulk samples. **a**, **e** The bright-field images of phase 1 and phase 2 in (Dy_0.25_Ho_0.25_Yb_0.25_Lu_0.25_)_2_Si_2_O_7_ sample. **b**, **f** The Scanning transmission electron microscopic high-angle annular dark-field (HAADF-STEM) images of phase 1 and phase 2 in (Dy_0.25_Ho_0.25_Yb_0.25_Lu_0.25_)_2_Si_2_O_7_. **c**, **g** The Fast Fourier transform (FFT) of the HAADF-STEM images of (Dy_0.25_Ho_0.25_Yb_0.25_Lu_0.25_)_2_Si_2_O_7_ in **b** and **f**, taken in the [$\bar{1}$12] zone axis and [100] zone axis, respectively. **d**, **h** The corresponding atomic-resolution EDS elemental maps for Dy, Ho, Yb and Lu of β-(Dy_0.25_Ho_0.25_Yb_0.25_Lu_0.25_)_2_Si_2_O_7_ and γ-(Dy_0.25_Ho_0.25_Yb_0.25_Lu_0.25_)_2_Si_2_O_7_. **i**, **m** The bright-field images of phase 1 and phase 2 in (La_x1_Ce_x2_Yb_x3_Lu_x4_)_2_Si_2_O_7_ sample. **j**, **n** The HAADF-STEM images of phase 1 and phase 2 in (La_x1_Ce_x2_Yb_x3_Lu_x4_)_2_Si_2_O_7_. **k**, **o** The Fast Fourier transform (FFT) of the HAADF-STEM images of (La_x1_Ce_x2_Yb_x3_Lu_x4_)_2_Si_2_O_7_ in **j**, **n**, taken in the [$\bar{1}\bar{1}$2] zone axis and [100] zone axis, respectively. **l**, **p** The corresponding atomic-resolution EDS elemental maps for La, Ce, Yb and Lu of β-(La_x1_Ce_x2_Yb_x3_Lu_x4_)_2_Si_2_O_7_ and G-(La_x1_Ce_x2_Yb_x3_Lu_x4_)_2_Si_2_O_7_. The blue circles in **a**, **e**, **i**, and **m** locate the observed grains in (Dy_0.25_Ho_0.25_Yb_0.25_Lu_0.25_)_2_Si_2_O_7_ and (La_x1_Ce_x2_Yb_x3_Lu_x4_)_2_Si_2_O_7_ samples that correspond to different phases (marked as “Phase 1” or “Phase 2”). The cyan circles and numbers in **c**, **g**, **k**, and **o** denote the Miller indices of corresponding patterns.

For the composition of (Dy_0.25_Ho_0.25_Yb_0.25_Lu_0.25_)_2_Si_2_O_7_ (Fig. 1b), the powders synthesized at 1550 °C contain dual-phase mixture of β-type solid solution as major phase, together with a decent amount of γ-type solid solution (indexed to γ-Y_2_Si_2_O_7_, PDF#42-0167). Quantitative phase analysis based on the Rietveld method (Fig. 2a) shows that, the β-phase accounts for 61.15 wt% in the mixture, whereas the γ-phase accounts for 38.85 wt%. Fabricating the sample via hot-pressing sintering method at 1650 °C results in decreased amount of β-phase down to 33.16 wt%, compensated by increased amount of γ-phase up to 66.84 wt% (Fig. 2b). After hot-pressed at 1860 °C for 2 h, the bulk material turns into a single-phase γ-type solid solution. According to the SEM-EDS results (Table 1), both β-phase and γ-phase in the 1650 °C hot-pressed bulk samples exhibit homogeneous RE elemental distributions, i.e., the atomic ratios of Dy:Ho:Yb:Lu are approximately 1:1:1:1. In addition, aberration-corrected scanning transmission electron microscopy (STEM) observations are performed on the 1650 °C hot-pressed (Dy_0.25_Ho_0.25_Yb_0.25_Lu_0.25_)_2_Si_2_O_7_ bulk sample (Fig. 3a–h), which enables the characterization of phase constitution and RE elemental distribution at the atomic scale. Figure 3a, b presents the bright-field image and STEM high-angle annular dark-field (HAADF) image of a chosen area in the sample (marked as “phase 1” in Fig. 3a). Figure 3c is the fast Fourier transform (FFT) patten of Fig. 3b, which is resolved to be β-(Dy_0.25_Ho_0.25_Yb_0.25_Lu_0.25_)_2_Si_2_O_7_ phase, taken in the [$\bar{1}$12] zone axis. Figure 3e–g present the results of another chosen area in the sample (marked as “phase 2” in Fig. 3e). Figure 3g is the FFT patten of phase 2 in Fig. 3f, which is resolved to be γ-(Dy_0.25_Ho_0.25_Yb_0.25_Lu_0.25_)_2_Si_2_O_7_ phase, taken in the [100] zone axis. Figure 3d, h present the selected compositional maps from EDS, respectively for β- and γ-phases, which directly visualizes the compositional homogeneity of RE elements (Dy, Ho, Yb and Lu) on the lattices of both β-(Dy_0.25_Ho_0.25_Yb_0.25_Lu_0.25_)_2_Si_2_O_7_ and γ-(Dy_0.25_Ho_0.25_Yb_0.25_Lu_0.25_)_2_Si_2_O_7_ phases. And, Supplementary Table 1 presents the atomic ratios of RE elements analyzed based on the TEM-EDS mapping results, which confirms the Dy:Ho:Yb:Lu being approximately 1:1:1:1.

For the composition of (Gd_0.25_Ho_0.25_Yb_0.25_Lu_0.25_)_2_Si_2_O_7_, Fig. 1c shows that the as-synthesized powders exhibit γ-(Gd_0.25_Ho_0.25_Yb_0.25_Lu_0.25_)_2_Si_2_O_7_ solid solution with very few β-(Gd_0.25_Ho_0.25_Yb_0.25_Lu_0.25_)_2_Si_2_O_7_; wherein the homogeneous distribution of RE elements, i.e., Gd:Ho:Yb:Lu being nearly 1:1:1:1, is confirmed by the SEM-EDS results (Table 1). Quantitative phase analysis (Fig. 2c) shows that the γ-phase accounts for 91.21 wt% in the mixture, whereas the β-phase accounts for 8.79 wt%. And, the small amount of β-phase is completely eliminated after hot-pressed at 1860 °C, due to the β → γ polymorphic transformation.

For the composition of (La_x1_Ce_x2_Yb_x3_Lu_x4_)_2_Si_2_O_7_ (x1 + x2 + x3 + x4 = 1), XRD results (Fig. 1d) shows that, both the as-synthesized powders and the hot-pressed bulk material are mixtures of β-type and G-type (indexed to G-Ce_2_Si_2_O_7_, PDF#48-0058) disilicates. Quantitative phase analysis (Fig. 2d, e) further reveals that the as-synthesized powders contain G-phase in 72.60 wt% and β-phase in 27.40 wt%, respectively; and, the phase composition remains almost unchanged after hot-pressed at 1650 °C for 2 h (G-phase in 72.15 wt% and β-phase in 27.85 wt%). This means that no phase transition reaction between β-type and G-type happens when we increase the sintering temperature or pressure, indicating the good phase stability of the mixture. SEM-EDS analysis (Table 1) shows obvious elemental segregation in both phases, i.e., the β-phase is enriched with Yb and Lu elements, whereas the G-phase is enriched with La and Ce elements. That is to say, it is easier to dope Yb and Lu (with smaller RE^3+^ radius) elements into the G-phase (La_2_Si_2_O_7_- or Ce_2_Si_2_O_7_-dominated); whereas highly difficult to dope La and Ce (with larger RE^3+^ radius) elements into the β-phase (Yb_2_Si_2_O_7_- or Lu_2_Si_2_O_7_-dominated). This could be understood from the perspective of atomic packing. When larger RE^3+^ cations are doped into a solvent dominated by smaller RE^3+^ cations, there is less room in the solvent polymorphic lattice to incorporate the larger solute polymorphic lattice, resulting in lower solubility; which is just the opposite if the doping sequence is reversed. In order to characterize the atomic distribution of RE elements in β-type and G-type (La_*x*1_Ce_*x*2_Yb_*x*3_Lu_*x*4_)_2_Si_2_O_7_, STEM observations are performed on the 1650 °C hot-pressed bulk sample. Figure 3i, m are the bright-field images of two different areas in the (La_*x*1_Ce_*x*2_Yb_*x*3_Lu_*x*4_)_2_Si_2_O_7_ sample, which are marked as “phase 1” and “phase 2” respectively. Figure 3j, n show the HAADF-STEM images of the two phases; and the corresponding FFT patterns in Fig. 3k, o are resolved to be β-(La_*x*1_Ce_*x*2_Yb_*x*3_Lu_*x*4_)_2_Si_2_O_7_ phase taken in the [$\bar{1}\bar{1}$2] zone axis, as well as G-(La_*x*1_Ce_*x*2_Yb_*x*3_Lu_*x*4_)_2_Si_2_O_7_ phase taken in the [100] zone axis, respectively. In addition, the selected atomic-resolution EDS elemental maps in Fig. 3l, p confirm the compositional uniformity of La, Ce, Yb and Lu elements in both β-(La_*x*1_Ce_*x*2_Yb_*x*3_Lu_*x*4_)_2_Si_2_O_7_ and G-(La_*x*1_Ce_*x*2_Yb_*x*3_Lu_*x*4_)_2_Si_2_O_7_ lattices, despite their non-stoichiometric distributions in different phases. The non-stoichiometric RE distributions could also be analyzed from the TEM-EDS mapping results, which are summarized in the Supplementary Table 1.

### Analysis on the average RE^3+^ cationic radius and the deviation

**Fig. 4:**
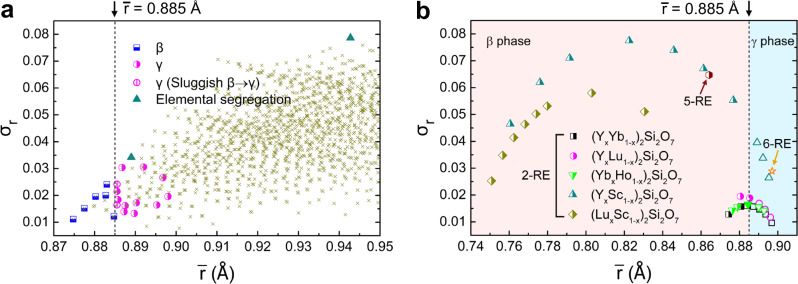
The average RE^3+^ radius ($\bar{r}$) and the deviations (*σ*_*r*_) for (*n*RE_*xi*_)_2_Si_2_O_7_ compounds. **a** The $\bar{r}$ versus *σ*_*r*_ diagram for (RE^I^_0.25_RE^II^_0.25_RE^III^_0.25_RE^IV^_0.25_)_2_Si_2_O_7_ compositions. The crosses represent the calculated $\bar{r}$ and *σ*_*r*_ parameters for all the possible RE^I^_0.25_RE^II^_0.25_RE^III^_0.25_RE^IV^_0.25_ combinations, wherein RE elements varies from the lanthanides (La~Lu) and Y elements. **b** The $\bar{r}$ versus *σ*_*r*_ diagram for some equimolar or non-equimolar (*n*RE_*xi*_)_2_Si_2_O_7_ (*n* = 2, 5 and 6; 0 ≤ *x*_*i*_ ≤ 1) materials, according to the works of ref. ^31, 41–47^. The red hexagon mark and the orange star represent the data for (Yb_0.2_Y_0.2_Lu_0.2_Sc_0.2_Gd_0.2_)_2_Si_2_O_7_ and (Gd_1/6_Tb_1/6_Dy_1/6_Tm_1/6_Yb_1/6_Lu_1/6_)_2_Si_2_O_7_ compounds, respectively; and “2-RE”, “5-RE” and “6-RE” represent the two-, five- and six-RE-principal-component disilicates. The β-γ boundary at $\bar{r}$~0.885 Å is marked in dash lines for emphasis.

Based on the above results, it would be interesting to summarize how the finally stabilized polymorphic types of the (RE^I^_0.25_RE^II^_0.25_RE^III^_0.25_RE^IV^_0.25_)_2_Si_2_O_7_ compositions, after phase formation and the possible phase transformation at sufficiently high temperatures, depend on the average RE^3+^ cationic radius ($\bar{r}$) as well as the deviation (*σ*_*r*_) of multiple RE elements. The statistics are summarized in Fig. 4a. Herein, the parameters $\bar{r}$ and *σ*_*r*_ are calculated as the average and standard deviation of the radius for each RE^3+^ cation, respectively, weighted by the molar fraction of the RE^3+^ cations in the target composition. Several interesting features are worth of noticing as follow.

Firstly, the β-phase-dominated zone rests in the far left of the Fig. 4a, corresponding to small $\bar{r}$ values; and as the $\bar{r}$ values increase, the γ-phase-dominated zone starts to take over. The potential boundary between the β- and γ-phase-dominated zone is estimated to be around $\bar{r}$~0.885 Å, which indicates that in order to design β-type (RE^I^_0.25_RE^II^_0.25_RE^III^_0.25_RE^IV^_0.25_)_2_Si_2_O_7_, the $\bar{r}$ parameter of the multi-RE^3+^ combination should be kept below 0.885 Å. Interestingly, this value coincides with our analysis on many other (*n*RE_*xi*_)_2_Si_2_O_7_ systems (Fig. 4b), including two-RE-principal-component disilicates, i.e., (Y_*x*_Yb_1−*x*_)_2_Si_2_O_7_^41^, (Y_*x*_Lu_1−__*x*_)_2_Si_2_O_7_^42,43^, (Yb_*x*_Ho_1−__*x*_)_2_Si_2_O_7_^44^, (Y_*x*_Sc_1−__x_)_2_Si_2_O_7_^45^, and (Lu_x_Sc_1−__*x*_)_2_Si_2_O_7_ (0 ≤ *x* ≤ 1)^46^ solid solutions; as well as five- and six-RE-principal-component disilicates, i.e., (Yb_0.2_Y_0.2_Lu_0.2_Sc_0.2_Gd_0.2_)_2_Si_2_O_7_^47^ and (Gd_1/6_Tb_1/6_Dy_1/6_Tm_1/6_Yb_1/6_Lu_1/6_)_2_Si_2_O_7_^31^ solid solutions. Detailed analysis on these compounds is presented in the Supplementary Note 2. Therefore, the $\bar{r}$~0.885 Å criterion, although herein extracted from the equimolar four-RE-principal-component (RE^I^_0.25_RE^II^_0.25_RE^III^_0.25_RE^IV^_0.25_)_2_Si_2_O_7_ systems, may also applies to other equimolar or non-equimolar (*n*RE_*xi*_)_2_Si_2_O_7_ (*n* denotes the total number of RE components, *x*_*i*_ denotes the molar fraction of the *i*th RE species, and $\mathop{\sum }\nolimits_{i=1}^{n}{x}_{i}=1$) systems. And, the fact that the $\bar{r}$~0.885 Å criterion is applicable to multi-RE^3+^ combinations including Sc^3+^, which has considerably smaller cationic radius (0.745 Å) as compared with the other RE^3+^ cations (ranging systematically from 0.861 to 1.032 Å for RE = lanthanides and yttrium), further verifies the good generality of this criterion.

Secondly, as indicated from Fig. 4a, the single-phase (RE^I^_0.25_RE^II^_0.25_RE^III^_0.25_RE^IV^_0.25_)_2_Si_2_O_7_ compounds could be formed only if the *σ*_*r*_ values are sufficiently small; while larger *σ*_*r*_ values will result in elemental segregation and phase separation, such as *σ*_*r*_ = 0.079 for (La_*x*1_Ce_*x*2_Yb_*x*3_Lu_*x*4_)_2_Si_2_O_7_ and *σ*_*r*_ = 0.034 for (Eu_*x*1_Tm_*x*2_Yb_*x*3_Lu_*x*4_)_2_Si_2_O_7_ (*x*1 + *x*2 + *x*3 + *x*4 = 1) systems. In fact, when multiple RE elements, which do not exhibit a certain polymorph of single-RE-principal-component RE_2_Si_2_O_7_ per se, are hosted into the target lattice, the so-called “imperfect isomorphism” will take place^9^. Hosting RE elements other than Yb and Lu into the β-type lattice is a good example of this situation. As such, the size mismatch and chemical difference amongst multiple RE^3+^ cations are expected to introduce extensive perturbations into the lattice, causing different phases to compete against each other during the formation of solid solutions. Small difference amongst multiple RE^3+^ cations could be tolerated in the lattice, and thus form single-phase multicomponent solid solution in a thermodynamically favored polymorphic type; whereas too large of the difference will cause elemental segregation and phase separation. Moreover, in order to approach the potential upper-limit of *σ*_*r*_ for β-type (RE^I^_0.25_RE^II^_0.25_RE^III^_0.25_RE^IV^_0.25_)_2_Si_2_O_7_, we calculate the $\bar{r}$ and *σ*_*r*_ parameters for all the possible RE^I^_0.25_RE^II^_0.25_RE^III^_0.25_RE^IV^_0.25_ combinations (i.e., in equimolar), wherein RE elements varies from the lanthanides and yttrium. The data are presented by cross-marks in the Fig. 4a. Particularly, for all the equimolar RE^I^_0.25_RE^II^_0.25_RE^III^_0.25_RE^IV^_0.25_ combinations falling in the β-phase-dominated zone, as bounded by the $\bar{r}$ < 0.885 criterion discussed above, their *σ*_*r*_ parameters are not high enough to reach the potential upper-limits; herein, the highest *σ*_*r*_ value corresponds to our synthesized β-(Tb_0.25_Tm_0.25_Yb_0.25_Lu_0.25_)_2_Si_2_O_7_ (*σ*_*r*_ = 0.024). This means that, pinpointing the potential upper-limit of *σ*_*r*_ parameter for β-type four-RE-principal-component RE_2_Si_2_O_7_ materials requires extending the compositional design into the non-equimolar cases.

Additional attention should be paid for the compositions lying in the vicinity of the β-γ boundary, such as $\bar{r}\,$ = 0.886 Å and *σ*_*r*_ = 0.016 for (Y_0.25_Ho_0.25_Tm_0.25_Lu_0.25_)_2_Si_2_O_7_, and $\bar{r}\,$ = 0.886 Å and *σ*_*r*_ = 0.024 for (Tb_0.25_Er_0.25_Yb_0.25_Lu_0.25_)_2_Si_2_O_7_. The trend of phase formation and transformation for these two compositions could be described as the formation of β-type (RE^I^_0.25_RE^II^_0.25_RE^III^_0.25_RE^IV^_0.25_)_2_Si_2_O_7_ solid solution followed by the occurrence of β → γ polymorphic transformation, only that the β → γ polymorphic transformation is quite sluggish. And therefore, higher reaction temperature, higher reaction pressure or longer holding time is needed to accelerate the β → γ transformation and ensure the stabilization of single-phase γ-type solid solutions for these compositions. Detailed discussions are presented in the Supplementary Note 3 and Supplementary Fig. 5.

### The configurational entropy of mixing

As indicated in the above results, the phase formation capability of β-type (RE^I^_0.25_RE^II^_0.25_RE^III^_0.25_RE^IV^_0.25_)_2_Si_2_O_7_ is closely related to the size or chemical mismatch of the multi-RE^3+^ combinations; and this could be translated into the level of randomness/disorders that the multicomponent system can tolerates, which echoes the concept of configurational entropy of mixing. In this section, we will probe into this issue from the perspective of thermodynamics.

**Fig. 5:**
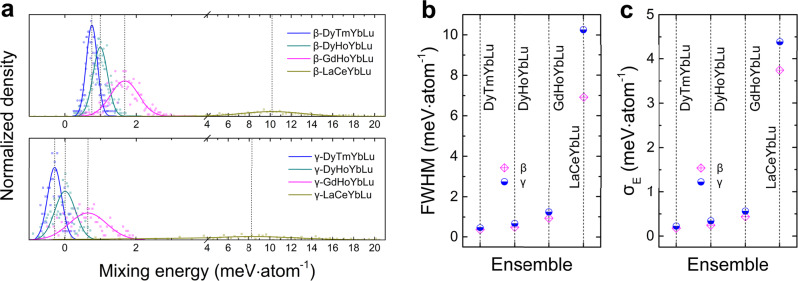
Statistical parameters for the Gaussian-fitted mixing energy (*E*_*i*_) distribution spectrum for β-type and γ-type ensembles. **a** The distribution of mixing energy (*E*_*i*_) for all the studied ensembles. The scatters are the normalized density for *E*_*i*_ analyzed by breaking the entire range of *E*_*i*_ values into small intervals Δ*E*, and then adding up the number of *E*_*i*_ that falls with each interval; herein, $\triangle E=\frac{5}{{N}^{'}}\left({E}_{{{{{{\rm{max }}}}}}}-{E}_{{{{{{\rm{min }}}}}}}\right)$ is used, and *N*′ is the total number of the sampled microstates in an ensemble. The solid lines are the Gaussian fitting of the *E*_*i*_ distribution spectrum. **b** The full width at half maximum (FWHM) of the Gaussian-fitted *E*_*i*_ distribution spectrum. **c** The standard deviation of mixing energy (*σ*_*E*_) for all the configurations in each ensemble.

The configuration ensembles of four prototype compositions are constructed, i.e., (Dy_0.25_Tm_0.25_Yb_0.25_Lu_0.25_)_2_Si_2_O_7_, (Dy_0.25_Ho_0.25_Yb_0.25_Lu_0.25_)_2_Si_2_O_7_, (Gd_0.25_Ho_0.25_Yb_0.25_Lu_0.25_)_2_Si_2_O_7_, and (La_0.25_Ce_0.25_Yb_0.25_Lu_0.25_)_2_Si_2_O_7_, and each includes hundreds of possible stochastically generated configurations, as illustrated in the Supplementary Fig. 6. Mind that there might be low energy barrier for β → γ polymorphic transformation, as suggested by their similarities in crystal structures and a collection of macroscopic properties; and therefore, both β-type and γ-type polymorphic ensembles are constructed. For each ensemble, the mixing energy (*E*_*i*_) for all configurations is calculated under the zero-temperature ground states, and their distributions are presented in Fig. 5a. In order to quantitatively analyze the features of the energy spread, the *E*_*i*_ distribution curves are fitted into the Gaussian function: $f\left(x\right)=a\cdot {{{{{\rm{exp }}}}}}\left(-\frac{{\left(x-b\right)}^{2}}{{c}^{2}}\right)$, wherein *a* determines the height of the peak of the distribution curve; *b* represents the position of the center of the peak; and *c* controls the width of the bell-shaped distribution curve, which is related to the full width at half maximum (FWHM) of the peak according to FWHM = $c\cdot 2\sqrt{{{{{{\rm{ln}}}}}}2}$. In this scheme, the FWHM measures the dispersity of *E*_*i*_ for all the microstates in the ensemble, and, therefore, can be used to depict the formation capability a multicomponent material. That is, a smaller FWHM (narrower *E*_*i*_ distribution spectrum) indicates lower energy barrier for the ergodicity of metastable configurations, and thus promotes high configurational randomness into the multicomponent system; whereas a larger FWHM (wider *E*_*i*_ distribution spectrum) indicates higher energy cost to access different configurations, and thus prefers elemental segregation or phase separation.

The FWHM parameter for each ensemble is analyzed in Fig. 5b. It is shown that the hypothetically constructed β-type and γ-type (La_0.25_Ce_0.25_Yb_0.25_Lu_0.25_)_2_Si_2_O_7_ ensembles have notably wider *E*_*i*_ distribution curve than the other ensembles, and the FWHM values are overwhelmingly higher than those of others, indicative of the least possibility to form homogeneous (La_0.25_Ce_0.25_Yb_0.25_Lu_0.25_)_2_Si_2_O_7_ solid solutions. In comparison, both β-type and γ-type polymorphic ensembles of the (Dy_0.25_Tm_0.25_Yb_0.25_Lu_0.25_)_2_Si_2_O_7_, (Dy_0.25_Ho_0.25_Yb_0.25_Lu_0.25_)_2_Si_2_O_7_, and (Gd_0.25_Ho_0.25_Yb_0.25_Lu_0.25_)_2_Si_2_O_7_ compositions show smaller FWHM values, ranging from 0.36121 to 1.23961 (Table 2), indicative of their relatively higher tendency of being formed. In fact, the FWHM parameter of the *E*_*i*_ distribution curve is in close relation with the standard deviation of mixing energy (*σ*_*E*_), which has been proposed as a descriptor of entropy in Sarker et al.’s work^14^. Herein, the *σ*_*E*_ values of each ensemble are also calculated and plotted in Fig. 5c, which shows similar trends as the FWHM parameter. Meanwhile, we find a positive correlation between the FWHM and *σ*_*r*_ parameters, i.e., the smaller *σ*_*r*_, the smaller FWHM (results are presented in Table 2) and the Pearson correlation coefficient (*ρ*) between the two parameters is calculated to be 99.54% for the β-type ensembles and 99.45% for the γ-type ensembles. This could be reasonably understood that large mismatch in the RE^3+^ cationic radius (stemming from the discrepancies in RE^3+^ electronic configurations) might increase the misfit of strain field for each RE^3+^ sublattice, and therefore significantly raise the energy fluctuations among the metastable configurations in the ensemble, making it energetically unfavorable to accommodate disorders into the designed polymorphic structures. And, it well explains why the formation of (RE^I^_0.25_RE^II^_0.25_RE^III^_0.25_RE^IV^_0.25_)_2_Si_2_O_7_ compounds with homogeneous RE elemental distribution requires the *σ*_*r*_ parameter to be sufficiently small.

**Table 2:** The FWHM and *σ*_*r*_ parameters for the studied ensembles

Composition	FWHM	*σ*_*r*_
β-(Dy_0.25_Tm_0.25_Yb_0.25_Lu_0.25_)_2_Si_2_O_7_	0.36121	0.01955
β-(Dy_0.25_Ho_0.25_Yb_0.25_Lu_0.25_)_2_Si_2_O_7_	0.47613	0.0215
β-(Gd_0.25_Ho_0.25_Yb_0.25_Lu_0.25_)_2_Si_2_O_7_	0.93006	0.03055
β-(La_0.25_Ce_0.25_Yb_0.25_Lu_0.25_)_2_Si_2_O_7_	6.91337	0.07867
γ-(Dy_0.25_Tm_0.25_Yb_0.25_Lu_0.25_)_2_Si_2_O_7_	0.46106	0.01955
γ-(Dy_0.25_Ho_0.25_Yb_0.25_Lu_0.25_)_2_Si_2_O_7_	0.67814	0.0215
γ-(Gd_0.25_Ho_0.25_Yb_0.25_Lu_0.25_)_2_Si_2_O_7_	1.23961	0.03055
γ-(La_0.25_Ce_0.25_Yb_0.25_Lu_0.25_)_2_Si_2_O_7_	10.25844	0.07867

Note: FWHM is defined as the full width at half maximum of the peaks in the mixing energy distribution spectrum, and *σ*_*r*_ is defined as the standard deviation of RE^3+^ cationic radius for a composition.

**Fig. 6:**
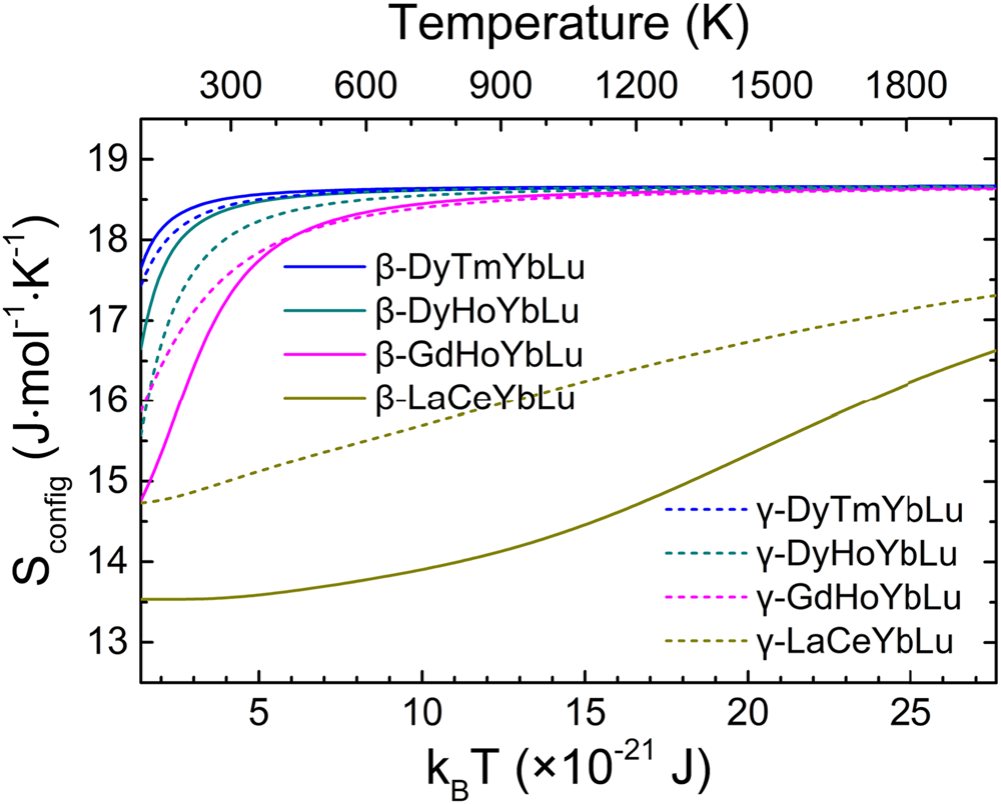
The configurational entropy of mixing for the β-type and γ-type ensembles. The calculated configurational entropy of mixing (*S*_config_) as a function of thermal-energy excitation (*k*_B_*T*) as well as the temperature (*T*) for each configuration ensemble.

In addition, by processing the *E*_*i*_ distribution profiles, the configurational entropy of mixing (*S*_config_) for each ensemble is calculated as a function of thermal-energy excitation (quantified by *k*_B_*T*, and *k*_B_ is the Boltzmann constant), which can be equated with the thermodynamic temperature (*T*) on the physical basis. Fundamentally, the *S*_config_~*k*_B_*T* (or *S*_config_~*T*) curve measures the number of microstates that a system may assume at a given amount of thermal-energy excitation beyond the ground state. As shown in Fig. 6, the *S*_config_ of (Dy_0.25_Tm_0.25_Yb_0.25_Lu_0.25_)_2_Si_2_O_7_, (Dy_0.25_Ho_0.25_Yb_0.25_Lu_0.25_)_2_Si_2_O_7_, and (Gd_0.25_Ho_0.25_Yb_0.25_Lu_0.25_)_2_Si_2_O_7_ systems converge to 18.70 J·mol^−1^·K^−1^. This is 18.8% lower than the ideal value of ${S}_{{{{{{\rm{config}}}}}}}^{{{{{{\rm{\infty }}}}}}}$ = 23.04 J·mol^−1^·K^−1^, which is estimated from ideal (RE^I^_0.25_RE^II^_0.25_RE^III^_0.25_RE^IV^_0.25_)_2_Si_2_O_7_ solid solution system, i.e., perfectly disordered crystalline with infinitely large lattice and totally random occupations on the doping sites. This deviation is probably due to the under-sampling of the configurational landscape prohibited by the size of the supercells used for simulation. Detailed explanation on the ${S}_{{{{{{\rm{config}}}}}}}^{{{{{{\rm{\infty }}}}}}}$ parameter is presented in the Supplementary Note 4. Therefore, if considered from the perspective of statistical mechanics and given sufficient thermal-energy excitations, the solid solutions of (Dy_0.25_Tm_0.25_Yb_0.25_Lu_0.25_)_2_Si_2_O_7_, (Dy_0.25_Ho_0.25_Yb_0.25_Lu_0.25_)_2_Si_2_O_7_ and (Gd_0.25_Ho_0.25_Yb_0.25_Lu_0.25_)_2_Si_2_O_7_ could be formed. By contrast, the *S*_config_ of (La_0.25_Ce_0.25_Yb_0.25_Lu_0.25_)_2_Si_2_O_7_ system is far from approaching the convergence at physically realizable temperatures, which means that it is highly impossible to form (La_0.25_Ce_0.25_Yb_0.25_Lu_0.25_)_2_Si_2_O_7_ solid solution with homogeneous RE distribution. What worthy of noticing is that, under the framework of statistical mechanics, the thermodynamic temperature “*T*” characterizes the thermal motion of molecules upon thermal excitation, and it is herein incorporated in the *S*_config_~*k*_B_*T* (or *S*_config_~*T*) curve to measure the amount of thermal-energy excitation that is needed for the ergodicity of metastable configurations in an ensemble. Any characteristic temperature extracted from the *S*_config_~*T* curve might not be directly compared to the real temperature that observed from the material synthesis process, because the latter is determined by many processing issues, such as the diffusion of elements, etc.

Another interesting point is that, the convergence tendency of *S*_config_~*k*_B_*T* (or *S*_config_~*T*) curve at relatively low thermal-energy excitation levels illustrates whether the β-type or γ-type crystal lattice would have higher possibility of being achieved upon forming homogeneous solid solution. Or rather, it qualitatively implies whether the configurational entropy of mixing has a positive or negative contribution to the β → γ polymorphic transformation. For instance, the (Dy_0.25_Ho_0.25_Yb_0.25_Lu_0.25_)_2_Si_2_O_7_ system shows *S*_config_(β-type) > *S*_config_(γ-type) within *T* < 300 K, which indicates that β-type lattice takes lower energy cost to accommodate sufficient disorder, and thus are more competitive to be formed than the γ-phase, on the *S*_config_ basis. In this case, the configurational entropy of mixing tends to procrastinate the β → γ polymorphic transformation. By contrast, the (Gd_0.25_Ho_0.25_Yb_0.25_Lu_0.25_)_2_Si_2_O_7_ system shows *S*_config_(γ-type) > *S*_config_(β-type), which indicates that the configurational entropy of mixing may accelerate the β → γ polymorphic transformation. Mind that besides the *S*_config_ we discussed here, the entropic driving force towards polymorphic transformation in rare-earth silicates comprises many other major contributions, such as the lattice vibrations.

Based on these results, we demonstrate that the *S*_config_ parameter (and its convergence trend with increased thermal-energy excitation) should be an effective descriptor to predict the formation capability of β-type (RE^I^_0.25_RE^II^_0.25_RE^III^_0.25_RE^IV^_0.25_)_2_Si_2_O_7_ solid solutions as well as the relative entropic contribution to the β → γ polymorphic transformation. In fact, as compared with other possible descriptors (such as the FWHM parameter discussed in this work and the *σ*_*E*_ parameter discussed in ref. ^14^, etc.), the *S*_config_ descriptor takes into account the relative ability of a multicomponent system to accommodate configurational randomness into different polymorphic types under accumulated thermal-energy excitation (beyond the ground state), and thus could properly capture the detailed thermodynamic differences between competing phases. In this respect, the *S*_config_ descriptor is expected to be useful in describing the phase formation capability of multicomponent materials showing polymorphism.

## Discussion

It is worthwhile to discuss some interesting implications of the current work on the design of (*n*RE_*xi*_)_2_Si_2_O_7_ materials with good high-temperature phase stability, considering their important applications in the TEBC systems. Hypothetically, the phase formation capability and high-temperature stability of (*n*RE_*xi*_)_2_Si_2_O_7_ materials could be depicted in a two-step scenario: whether a target polymorph can be formed in the designed stoichiometry and shows homogeneous RE elemental distribution; and whether the synthesized polymorph could be stabilized up to higher temperature ranges. As illustrated in this work, these two aspects are highly related with the $\bar{r}$ and *σ*_*r*_ parameters.

For one thing, the results in this work demonstrate that the formation of a multicomponent system could be conceptualized as the ergodicity of metastable configurations in an ensemble, which contains all the possible configurations resulted from the randomized distributions of multiple doping RE elements. Specifically, for a designed composition to form homogeneous solid solution, most of the metastable configurations should have appreciable probabilities of being formed under a certain amount of excitation, allowing for high configurational randomness into the system; that is to say, lower energy is needed to drive the system towards becoming a homogeneous solid solution. By contrast, if achieving similar level of randomness require extremely high level of thermal-energy excitation, then the system will turn into elemental segregation or phase separation. And, the extent to which the configurational randomness could be tolerated in a specific RE_2_Si_2_O_7_ polymorph is found to be manifested in the *σ*_*r*_ parameter: the smaller *σ*_*r*_ value, the higher propensity to form homogeneous solid solution.

**Fig. 7:**
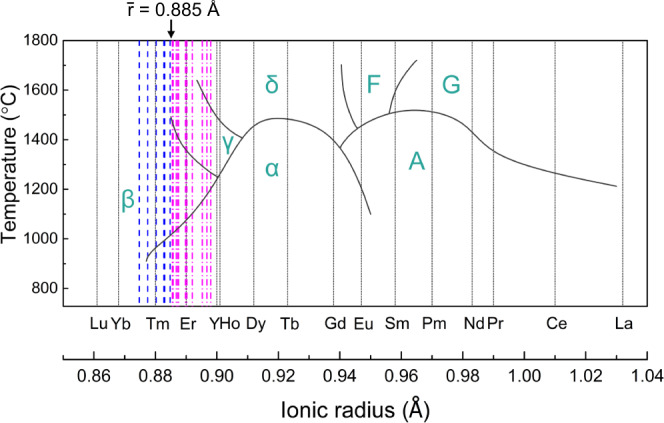
The polymorphic formation and transformation diagram for RE_2_Si_2_O_7_ materials. The cyan letters (α, β, γ, δ, A, F, and G) represent the seven different polymorphs of single-RE-principal-component RE_2_Si_2_O_7_ materials. The results for the (RE^I^_0.25_RE^II^_0.25_RE^III^_0.25_RE^IV^_0.25_)Si_2_O_7_ compositions synthesized in this work are added for comparison. The blue dash lines and the pink dash-dot lines mark the average RE^3+^ radius of the compositions that are finally stabilized at β and γ polymorphs, respectively, at high temperatures. The β-γ boundary located at the average RE^3+^ radius of $\bar{r}$~0.885 Å is marked with arrow for emphasis. The stability range for single-RE-principal-component RE_2_Si_2_O_7_ is reproduced based on Ref. ^9^, Bondar, I.A., Rare-earth silicates, Ceramics International, 8, 83–89, Copyright (1982), with permission from Elsevier.

For another, it will be interesting to evaluate how the formation capability of β-type (*n*RE_*xi*_)_2_Si_2_O_7_ compounds as well as their phase stability up to high temperature ranges refreshes the well-established knowledge of single-RE-principal-component RE_2_Si_2_O_7_ systems. Figure 7 presents the polymorphic formation and transformation diagram of all the single-RE-principal-component RE_2_Si_2_O_7_ compounds, as a function of the RE^3+^ cationic radius and temperature^9^. Also included in the diagram are the $\bar{r}$ parameters of the (RE^I^_0.25_RE^II^_0.25_RE^III^_0.25_RE^IV^_0.25_)_2_Si_2_O_7_ materials synthesized in this work. Except for the (La_*x*1_Ce_*x*2_Yb_*x*3_Lu_*x*4_)_2_Si_2_O_7_ and (Eu_*x*1_Tm_*x*2_Yb_*x*3_Lu_*x*4_)_2_Si_2_O_7_ (*x*1 + *x*2 + *x*3 + *x*4 = 1) system, which cannot form solid solutions with homogeneous and iso-stoichiometric distribution of rare earth elements, the polymorphic types that the (RE^I^_0.25_RE^II^_0.25_RE^III^_0.25_RE^IV^_0.25_)_2_Si_2_O_7_ compounds can be formed and stabilized are compatible with the phenomenological rule for the single-RE-principal-component RE_2_Si_2_O_7_ compounds. Specifically, the boundary between the β- and γ-phase-dominated zone is, for the first time, quantitatively recognized as $\bar{r}$~0.885 Å, below which the corresponding compositions should have β-type structure and show good stability up to high temperatures. The compatible trend of phase formation for single- and multi-RE-principal-component RE_2_Si_2_O_7_ compounds, in terms of the individual or average RE^3+^ cationic radius, is quite enlightening. Given that the (*n*RE_*xi*_)_2_Si_2_O_7_ solid solutions, regardless of the total number of RE components as well as their molar ratio, is virtually formed into a specific polymorphic structure with randomized distribution on the RE sites (governed by sufficiently small *σ*_*r*_ parameter as discussed above), it is then understandable that the interatomic environments and the polymorphic structures should be controlled by the effective cationic field strength of the system. And, the $\bar{r}$ parameter exhibits a direct measurement of the effective cationic field strength for the (*n*RE_*xi*_)_2_Si_2_O_7_ systems, under the premise that the mixed RE^3+^ cations show the similar valency. Our test from DFT calculations shows that the interatomic force and the bond length for Si-O and RE-O bonds in the β-(Er_0.25_Tm_0.25_Yb_0.25_Lu_0.25_)_2_Si_2_O_7_ lattice matches well with the average of the four parent β-RE_2_Si_2_O_7_ (RE = Er, Tm, Yb and Lu) lattices (Supplementary Fig. 7). This implies that the mixing of multiple RE elements tends to create an overall lattice environment that matches with the situation where the RE sites are occupied by a pseudo atom having the average bonding characteristics of the mixing RE atoms, on the premise of them showing similar bonding environments in the respective parent RE_2_Si_2_O_7_ lattices (i.e., reflected by small *σ*_*r*_ parameter). These results indicates that the polymorphic formation and transformation diagram of single-RE-principal-component RE_2_Si_2_O_7_ compounds could be reliably referenced to guide the design of (*n*RE_*xi*_)_2_Si_2_O_7_ materials in any of the seven polymorphic types. In addition, we have preliminarily tested that the $\bar{r}$-and-*σ*_*r*_ criteria could be generalized to predict the phase formation and stabilization of many equimolar or non-equimolar (*n*RE_*xi*_)_2_Si_2_O_7_ materials, and have led to successful synthesis of some new materials, such as β-(Dy_0.1_Tm_0.9_)_2_Si_2_O_7_, β-(Er_0.4_Tm_0.3_Yb_0.2_Lu_0.1_)_2_Si_2_O_7_, γ-(Dy_0.3_Tm_0.7_)_2_Si_2_O_7_, γ-(Gd_0.2_Ho_0.3_Yb_0.4_Lu_0.1_)_2_Si_2_O_7_, and γ-(Gd_1/6_Tb_1/6_Dy_1/6_Tm_1/6_Yb_1/6_Lu_1/6_)_2_Si_2_O_7_, etc. (see Supplementary Fig. 8 for examples).

At last, we remind that one prime goal of designing (*n*RE_*xi*_)_2_Si_2_O_7_ materials is to achieve the mix-and-match of several key RE ingredients, in the hope of synergistic optimization on the mechanical, thermal and corrosion-resistant properties, while maintaining good high-temperature phase stability. Based on this work, a two-step strategy could be proposed to guide the compositional design of β-type (*n*RE_*xi*_)_2_Si_2_O_7_ materials. First, several key RE ingredients should be specially included, from the property-oriented considerations; while other RE elements should be used as the adjustive RE ingredients, by resorting to the $\bar{r}$-and-*σ*_*r*_ criteria. And then, the optimal composition could be resolved by adjusting the number of RE elements and/or their molar ratios to tailor the *σ*_*r*_ and $\bar{r}$ values, i.e., by minimizing the *σ*_*r*_ of the multi-RE^3+^ combination to allow for sufficient configurational randomness into the solid solution; and at the same time tuning the $\bar{r}$ values into the β-phase-dominated zone (i.e., $\bar{r}\,$ < 0.885 Å) of the polymorphic formation and transformation diagram of RE_2_Si_2_O_7_. Based on this strategy, the compositional design of β-type (*n*RE_*xi*_)_2_Si_2_O_7_ materials is being pushed from the conventional trial-and-error manner up into a quantitative level. This design strategy will be applied and extensively tested in our future works, which may hopefully lead to the efficient screening of (*n*RE_*xi*_)_2_Si_2_O_7_ materials with desired properties.

In summary, the phase formation capability of twenty-one (RE^I^_0.25_RE^II^_0.25_RE^III^_0.25_RE^IV^_0.25_)Si_2_O_7_ (RE = Y, La, Ce, Eu, Gd, Tb, Dy, Ho, Er, Tm, Yb, and Lu) compounds, as representatives of the (*n*RE_*xi*_)_2_Si_2_O_7_ materials, are investigated via experimental and high-throughput density functional theory calculations. It is found that the phase formation capability of β-type (RE^I^_0.25_RE^II^_0.25_RE^III^_0.25_RE^IV^_0.25_)Si_2_O_7_ materials is governed by low energy barrier for the ergodicity of metastable configurations in an ensemble and thus accommodate sufficient configurational randomness of multiple RE^3+^ cations in β-type structure, while resist the β → γ polymorphic transformation. Interestingly, the phase formation and stabilization are jointly influenced by the average RE^3+^ radius ($\bar{r}$) as well as the deviations (*σ*_*r*_) of different RE^3+^ combinations. Specifically, β-type (RE^I^_0.25_RE^II^_0.25_RE^III^_0.25_RE^IV^_0.25_)Si_2_O_7_ materials could be formed and further stabilized up to high temperature ranges when satisfying the criteria of (1) $\bar{r}\,$ < 0.885 Å, as well as (2) sufficiently small *σ*_*r*_ values. Moreover, the configurational entropy of mixing (*S*_config_) is demonstrated as an effective descriptor to predict the formation capability of β-type (RE^I^_0.25_RE^II^_0.25_RE^III^_0.25_RE^IV^_0.25_)Si_2_O_7_ materials, which is able to quantify the propensity of each hypothetical composition to form β-type solid solutions with homogeneous RE elemental distribution, and simultaneously predicts whether the configurational entropy of mixing has a positive or negative contribution to the β → γ polymorphic transformation. Finally, a guideline is proposed to precisely control the phase formation capability and high-temperature stability of β-type (*n*RE_*xi*_)_2_Si_2_O_7_ materials, which pushes the frontier of compositional design of such materials to a quantitative scheme. This work is expected to accelerate the design and fabrication of β-type (*n*RE_*xi*_)_2_Si_2_O_7_ materials with tailored compositions and controlled polymorphic phases, which are highly demand for the application of TEBC system.

## Methods

### Experimental

Commercially available powders of Y_2_O_3_, La_2_O_3_, CeO_2_, Eu_2_O_3_, Gd_2_O_3_, Tb_4_O_7_, Dy_2_O_3_, Ho_2_O_3_, Er_2_O_3_, Tm_2_O_3_, Yb_2_O_3_, and Lu_2_O_3_ (Rear-Chem. Hi-Tech. Co. Ltd., Huizhou, China), and SiO_2_ (Sinopharm Chemical Reagent Co. Ltd., Shanghai, China) were used as starting materials to fabricate the (*n*RE_*xi*_)_2_Si_2_O_7_ samples. For each target composition, rare-earth oxides and SiO_2_ powders were mixed according to the stoichiometry of (RE^I^_0.25_RE^II^_0.25_RE^III^_0.25_RE^IV^_0.25_)_2_Si_2_O_7_, and then ball milled for 24 h in a Si_3_N_4_ jar with Si_3_N_4_ balls and ethanol solution. The obtained slip was dried at 60 °C for 24 h and then passed through a 120-mesh sieve. Pressure-less synthesis method was adopted to synthesize the (*n*RE_*xi*_)_2_Si_2_O_7_ powders at 1550 °C for 6 h or 1800 °C for 1 h. To promote the concerned β → γ polymorphic transformation by accelerating the mass transfer in the solid-state synthesis reaction, some (*n*RE_*xi*_)_2_Si_2_O_7_ samples were additionally hot-pressed in a BN-coated graphite mold at 1650 °C, 1800 °C, 1860 °C or 1900 °C for 1 h or 2 h under the pressure of 30 MPa. The phase compositions of (*n*RE_*xi*_)_2_Si_2_O_7_ samples were determined using an X-ray diffractometer (D/max-2400, Rigaku, Tokoy, Japan). Rietveld refinement method was used for quantitative phase analysis using the General Structural Analysis System (GSAS) program.^48,49^ Phase identification and rare-earth elements distribution of bulk samples were observed with a SEM (SUPRA 35, Zeiss, Oberkochen, Germany) equipped with an energy dispersive spectrometer (INCAx-sight, Oxford Instruments, Oxford, UK), and the point analysis for each phase was repeated for at least 7 times. The atomic distributions of rare earth elements in (*n*RE_*xi*_)_2_Si_2_O_7_ samples were characterized by a double aberration-corrected scanning transmission electron microscope (STEM) (Titan Themis Z, Thermo Fisher Scientific, Waltham, USA) equipped with high angle annular dark field (HAADF) detector and energy dispersive X-ray spectroscopy (EDS) systems.

### Calculation of configurational entropy

Under the framework of equilibrium statistical mechanics, the concept of entropy for a macroscopic variable could be interpreted as measuring the extent to which the probability of the system is spread out over different possible microstates. In this study, the (RE^I^_0.25_RE^II^_0.25_RE^III^_0.25_RE^IV^_0.25_)_2_Si_2_O_7_ solid solution with random occupations of RE^3+^ cations on its sublattice sites could be understood as the macrostate; while the possible metastable configurations could be understood as the microstates. The thermodynamics of the macrostate could be derived from the partition function, which encodes how the probabilities are partitioned among different microstates based on their individual configurational energies. The partition function could be written in the formula for the canonical ensemble^38,50^:

1 $$Z=\mathop{\sum }\limits_ {i=1}^{N}{{{{{\rm{exp }}}}}}\left(-\frac{{E}_{i}}{{k}_{{{{{{\rm{B}}}}}}}T}\right)$$

Herein, *i* is the index for the microstates of the system; *N* is the total number of the microstates; *k*_B_ is the Boltzmann constant; and *T* is the absolute temperature. *E*_*i*_ is the energy for the *i*th microstate, derived from the standard DFT total energy calculations (at temperature of *T* = 0 K):

2 $${E}_{i}={E}_{4{{{{{\rm{mix}}}}}}}-\frac{1}{4}{E}_{{{{{{{\rm{RE}}}}}}}^{{{{{{\rm{I}}}}}}}}-\frac{1}{4}{E}_{{{{{{{\rm{RE}}}}}}}^{{{{{{\rm{II}}}}}}}}-\frac{1}{4}{E}_{{{{{{{\rm{RE}}}}}}}^{{{{{{\rm{III}}}}}}}}-\frac{1}{4}{E}_{{{{{{{\rm{RE}}}}}}}^{{{{{{\rm{IV}}}}}}}}$$

where *E*_4mix_ is the total energy of the (RE^I^_0.25_RE^II^_0.25_RE^III^_0.25_RE^IV^_0.25_)_2_Si_2_O_7_ unit cell; *E*_RE_ (with superscript I−IV) is the total energy of the corresponding single-RE-principal-component RE_2_Si_2_O_7_, in the same polymorphic structure as the multicomponent material. Herein, the calculated *E*_*i*_ could be understood as the energy of mixing, which interprets the procedure of several single-RE-principal-component RE_2_Si_2_O_7_ mixed into a multi-RE-principal-component system through random assignment of the RE atoms onto the RE lattice sites. Then, the configurational energy for the macrostate could be written as the ensemble average of *E*_*i*_, which is the sum of energies for the microstates weighted by their probabilities:

3 $$\left\langle E\right\rangle=\frac{1}{Z}\mathop{\sum }\limits_{i=1}^{N}{E}_{i}{{{{{\rm{exp }}}}}}\left(-\frac{{E}_{i}}{{k}_{{{{{{\rm{B}}}}}}}T}\right)$$

And, the free energy raised from configurational disorders is given by:

4 $$F=-{k}_{{{{{{\rm{B}}}}}}}T{{{{{\rm{ln}}}}}}Z$$

In practice, it is computationally challenging to model the ensemble of all possible configurations for the multicomponent materials. Instead, the ensemble average could be performed over a representative subset of the whole microstate populations, provided that the subset extensively samples the energy landscape of all the microstates, and thus lead to statistically converged thermodynamic properties with respect to those of the whole population. This restriction is expressed by:

5 $$\frac{Z}{N}=\frac{Z^{\prime} }{N^{\prime} }$$

Herein, *N*’ is the total number of the sampled microstates; and *Z*′ is their partition function. Imposing the Eq. (5) into the Eq. (4) gives:

6 $$F=-{k}_{{{{{{\rm{B}}}}}}}T\,{{{{{\mathrm{ln}}}}}}\left(N\cdot \frac{Z^{\prime} }{N^{\prime} }\right)=-{k}_{{{{{{\rm{B}}}}}}}T\,{{{{{\mathrm{ln}}}}}}\,N-{k}_{{{{{{\rm{B}}}}}}}T\,{{{{{\mathrm{ln}}}}}}\,\frac{{\sum }_{i=1}^{N^{\prime} }\exp \left(-\frac{{E}_{i}}{{k}_{{{{{{\rm{B}}}}}}}T}\right)}{N^{\prime} }$$

Finally, the configurational entropy is derived from:

7 $${S}_{{{{{{\rm{config}}}}}}}=\left(\left\langle E\right\rangle -F\right)/T$$

It should be noticed that, in general, the entropy of materials comprises contributions from configuration, vibration, electronic excitation, magnetism, etc. This model mainly deals with the configurational entropy of mixing, as it is expected to have significant effect on the formation of multicomponent ceramics. Such theoretical framework has been successfully adopted in Anand et al.’s work to interpret the thermodynamics of high-entropy oxides.^38^ Alternatively, one could also examine the energy spread of a system by calculating the formation energy for every metastable configuration in the ensemble, such as using the RE_2_O_3_ and SiO_2_ as reference states in the Eq. (2). Results are discussed in the Supplementary Note 5 and Supplementary Fig. 9. Such modification will not change the calculated *S*_config_, as it is derived from the dispersive features of the energy spread.

The available configurations for the multicomponent solid solution are generated by employing the special quasirandom structures (SQS) generation code implemented in the Alloy Theoretic Automated Toolkit (ATAT) package^51^, which allows for the simulation of disordered crystallines by sampling on supercells with varied shapes and randomized occupations on the RE cationic sites (Supplementary Fig. 6a). Supercells with 88 lattice sites are used to construct the β-type and γ-type (RE^I^_0.25_RE^II^_0.25_RE^III^_0.25_RE^IV^_0.25_)_2_Si_2_O_7_ systems, corresponding to four times of the minimum cell size necessary to reproduce the required stoichiometry. The configuration ensembles are constructed to include 558 unique configurations for the β-type (RE^I^_0.25_RE^II^_0.25_RE^III^_0.25_RE^IV^_0.25_)_2_Si_2_O_7_, and 319 for the γ-type structures. Details on the stochastic generation of configuration ensembles, as well as the convergence test on the size of configuration ensembles, are presented in the Supplementary Note 6 and Supplementary Fig. 10.

### Computational details

Density functional theory (DFT) calculations is performed using the Vienna Ab Initio Simulation Package (VASP)^52^. The interactions between core electrons and valence electrons are described by the projector augmented wave (PAW) method^53^. The Perdew-Burke-Ernzerhof (PBE)^54^ form of the generalized gradient approximation (GGA) is used to formulate the electronic exchange correlations functional. The energy cut-off for plane-wave basis is set as 600 eV, which has been tested to give accurate prediction on the energy spread of configuration ensembles (see Supplementary Note 7 and Supplementary Fig. 11 for the results of convergence test). The Brillouin zone (BZ) is sampled using the Γ-centered Monkhorst-Pack^55^ *k*-mesh in the resolution of 2π × 0.03 Å^−1^. For geometric optimization, the lattice parameters and internal atomic positions are fully relaxed, and the convergence criteria are set as 1×10^−7^ eV for the total energy and 5 × 10^−3^ eV/Å for the ionic Hellmann–Feynman forces, respectively.

## Supplementary information

Supplementary Information

## Data Availability

The raw data generated in this study have been deposited in the Materials Cloud, which can be accessed from 10.24435/materialscloud:7e-ar,^56^ and are available from the corresponding author upon request.

## Code availability

Vienna Ab Initio Simulation Package (VASP) is available at www.vasp.at. The custom codes used in this work have been deposited in Zenodo, which can be accessed from 10.5281/zenodo.7580484,^57^ and are available from the corresponding author upon request.
