# Supplementary material for: Phase formation capability and compositional design of β-phase multiple rare-earth principal component disilicates
Source: Nat Commun. 2023 Mar 8;14:1275. doi: 10.1038/s41467-023-36947-6 (PMC9992687; doi:10.1038/s41467-023-36947-6)
Supplement: Supplementary file 1 — Supplementary Information [file 41467_2023_36947_MOESM1_ESM.pdf]

Supplementary Information for

Phase formation capability and compositional design of β-phase multiple rare-earth principal component disilicates

Yixiu Luo[1], Luchao Sun[1]*, Jiemin Wang[1], Tiefeng Du[1], Cui Zhou[1,2], Jie Zhang[1], Jingyang Wang[1]*

[1]Shenyang National Laboratory for Materials Science, Institute of Metal Research, Chinese Academy of Sciences, Shenyang 110016, China

[2]School of Materials Science and Engineering, University of Science and Technology of China, Shenyang 110016, China

These authors jointly supervised this work: Luchao Sun, Jingyang Wang

*Email: lcsun@imr.ac.cn Tel: +86-24-23971449

jywang@imr.ac.cn Tel: +86-24-23971762

Supplementary Notes

Supplementary Note 1: The rationale of choosing the model compositions

In this study, twenty-one $(RE^{I}_{0.25}RE^{II}_{0.25}RE^{III}_{0.25}RE^{IV}_{0.25})_2Si_2O_7$ (RE = Y, La, Ce, Eu, Gd, Tb, Dy, Ho, Er, Tm, Yb, and Lu) compositions are chosen as model systems, and the rationale is threefold. Firstly, the polymorphism of single-RE-principal-component $RE_2Si_2O_7$ materials is related to the radius of RE^{3+} cations [1]. Listing in the sequence of decreased RE^{3+} cationic radius, $La_2Si_2O_7$ and $Ce_2Si_2O_7$ exhibit A-type polymorph at lower temperature and G-type at higher temperature; $Eu_2Si_2O_7$ exhibit α-type at lower temperature, and δ-type or F-type at higher temperature; $Gd_2Si_2O_7$, $Tb_2Si_2O_7$ and $Dy_2Si_2O_7$ exhibit α-type at lower temperature and δ-type at higher temperature; $Ho_2Si_2O_7$ exhibits $\beta \rightarrow \gamma \Leftrightarrow \delta$ transformation with increased temperature; $Tm_2Si_2O_7$ exhibits α-type at lower temperature and β-type at higher temperature; $Yb_2Si_2O_7$ and $Lu_2Si_2O_7$ are stabilized at β-type structure up to the melting point; and $Y_2Si_2O_7$ exhibits seven different polymorphs. Intuitively, RE elements with smaller cationic radius (i.e., Yb and Lu) should be necessary ingredients for the formation of β-type multi-RE-principal-component $(n\text{RE}_{xi})_2Si_2O_7$ materials. Based on this consideration, the twenty-one different $RE^{I}RE^{II}RE^{III}RE^{IV}$ combinations are designed to include either Yb or Lu, or both; while the other RE elements are sampled across the lanthanides and yttrium. Secondly, by incorporating RE elements that do not exhibit β-type single-RE-principal-component $RE_2Si_2O_7$ per se into the $RE^{I}RE^{II}RE^{III}RE^{IV}$ combinations, we might be able to massively expand the compositional space of

β-$RE_2Si_2O_7$ polymorph, and at the same time pinpoint the extent to which the randomness/disorder brought by these "alien" RE elements will be tolerated in the β-type lattice. Thirdly, the twenty-one designed compositions show systematic discrepancies in the average RE^{3+} cationic radii as well as their deviations, and hence enables detecting how these factors might affect the formation capability of $(n\mathrm{RE}_{x_i})_2Si_2O_7$ materials. One thing to notice is that, despite the good stability of β-$Sc_2Si_2O_7$ up to the melting point, the Sc element is not included in the compositional design, mainly due to considerably smaller Sc^{3+} cationic radius (0.745 Å) as compared with the other RE^{3+} cations (ranging systematically from 0.861 Å to 1.032 Å for RE = lanthanides and yttrium).

Supplementary Note 2: Phase formation of multi-RE-principal-component $(n\mathrm{RE}_{x_i})_2Si_2O_7$ (n=2, 5 and 6; $0 \leq x_i \leq 1$) solid solutions

Becerro et al. have studied the phase formation and stabilization of $(Lu_xSc_{1-x})_2Si_2O_7$, $(Y_xYb_{1-x})_2Si_2O_7$, $(Y_xSc_{1-x})_2Si_2O_7$, and $(Y_xLu_{1-x})_2Si_2O_7$ ($0 \leq x \leq 1$) systems [2-6]. It has been found that all these compositions can form complete solid solutions. Specifically, the $(Lu_xSc_{1-x})_2Si_2O_7$ ($0 \leq x \leq 1$) systems show stable β-phase up to 1500°C, due to the isostructure of β-$Lu_2Si_2O_7$ and β-$Sc_2Si_2O_7$. For the $(Y_xYb_{1-x})_2Si_2O_7$, $(Y_xSc_{1-x})_2Si_2O_7$, and $(Y_xLu_{1-x})_2Si_2O_7$ systems, the β-, γ- and δ-phase emerges successively with increased content of Y and increased temperature; and the β-phase shows good stability up to 1700°C in the range of $0 \leq x \leq 0.55$, $0 \leq x \leq 0.625$ and $0 \leq x \leq 0.85$ for the three systems, respectively. Based on these experimental data, the average RE^{3+} cationic radii ($\bar{r}$) as well as the deviations (σ_r) of these materials are

summarized in Fig. 4b of the main manuscript. It is found that the potential boundary between β- and γ-phase-dominated zone is approximated to be $\bar{r}$~0.885 Å, in consistent with our findings from the $(RE^{I}_{0.25}RE^{II}_{0.25}RE^{III}_{0.25}RE^{IV}_{0.25})_2Si_2O_7$ (RE = lanthanides and yttrium) systems. Meanwhile, the maximum σ_r values for these systems are calculated to be 0.016 and 0.020 for the β-$(Y_xYb_{1-x})_2Si_2O_7$ and β-$(Y_xLu_{1-x})_2Si_2O_7$ solid solutions, both falling in the tolerance range extracted from the β-type $(RE^{I}_{0.25}RE^{II}_{0.25}RE^{III}_{0.25}RE^{IV}_{0.25})_2Si_2O_7$ compounds (Fig. 4a of the main manuscript); on the other hand, the maximum σ_r values are 0.058 and 0.078 for the β-$(Lu_xSc_{1-x})_2Si_2O_7$ and β-$(Y_xSc_{1-x})_2Si_2O_7$ systems, respectively. Herein, the higher σ_r values for the Sc-contained systems is a reasonable consequence of the obviously smaller cationic radius of Sc^{3+} (0.745 Å) as compared with the other RE^{3+} cations (ranging systematically from 0.861 Å to 1.032 Å for RE = lanthanides and yttrium). Therefore, we deduce that the potential upper-limit for the σ_r parameter should be different, depending on whether or not to include the Sc^{3+} cation in the compositional design.

Meanwhile, Lv et al. have reported the phase composition of $(Yb_{1-x}Ho_x)_2Si_2O_7$ (x = 0, 1/4, 1/3, 1/2, 2/3, 3/4, 1) [7], wherein the powder samples were synthesized via pressure-less sintering method at 1550°C, and the bulk samples were fabricated via Spark Plasma Sintering method at 1550°C with a holding time of 10 min. It is found that the synthesized solid solutions show pure β-phase at x = 1/4 and 1/3; whereas pure γ-phase at x = 2/3 and 3/4; and the composition of $(Yb_{1/2}Ho_{1/2})_2Si_2O_7$ is identified to be a mixture of β-phase along with a small amount of γ-phase. As

summarized in Fig. 4b in the main manuscript, the σ_r parameters of these compositions are sufficiently small (range from 0.014 to 0.017), which is expected to promote the formation of stoichiometric solid solutions. And, the boundary between β- and γ-phase-dominated zone is also approximated as $\bar{r}$~0.885 Å. Particularly, the $\bar{r}$ parameter of $(Yb_{1/2}Ho_{1/2})_2Si_2O_7$ ($\bar{r}$=0.885 Å) lies exactly in the vicinity of the β-γ boundary, which should result in the sluggish β→γ polymorphic transformation and thus coexistence of β- and γ-phase in this composition.

Furthermore, experimental observations on the phase formation of high-than-four-RE principal-component $(nRE_{xi})_2Si_2O_7$ disilicates are available for $(Yb_{0.2}Y_{0.2}Lu_{0.2}Sc_{0.2}Gd_{0.2})_2Si_2O_7$ [8] and $(Gd_{1/6}Tb_{1/6}Dy_{1/6}Tm_{1/6}Yb_{1/6}Lu_{1/6})_2Si_2O_7$ [9], which are reported to be stable β-phase and γ-phase, respectively, up to the melting point. As shown in Fig. 4b in the main manuscript, the trend of $\bar{r}$ and σ_r parameters for these two compounds are analyzed to be consistent with the other $(nRE_{xi})_2Si_2O_7$ systems.

Supplementary Note 3: Sluggish β→γ polymorphic transformation in $(Y_{0.25}Ho_{0.25}Tm_{0.25}Lu_{0.25})_2Si_2O_7$ and $(Tb_{0.25}Er_{0.25}Yb_{0.25}Lu_{0.25})_2Si_2O_7$

The composition of $(Y_{0.25}Ho_{0.25}Tm_{0.25}Lu_{0.25})_2Si_2O_7$ has $\bar{r}$=0.886 Å and σ_r=0.016, and $(Tb_{0.25}Er_{0.25}Yb_{0.25}Lu_{0.25})_2Si_2O_7$ has $\bar{r}$=0.886 Å and σ_r=0.024, both lie in the vicinity of the β-γ boundary. For the $(Y_{0.25}Ho_{0.25}Tm_{0.25}Lu_{0.25})_2Si_2O_7$ compound, the XRD patterns (Supplementary Fig. 3a) combined with Rietveld refinement (Supplementary Fig. 5a, b) reveal that the as-synthesized (1550°C) powder is a dual-phase mixture containing 92.26 wt% β-phase and 7.74 wt% γ-phase solid solutions; which turn into the mixture of 28.98 wt% β-phase and 71.02 wt% γ-phase

after hot-pressing at 1800°C, due to the β→γ polymorphic transformation; and finally, single-phase γ-$(Y_{0.25}Ho_{0.25}Tm_{0.25}Lu_{0.25})_2Si_2O_7$ solid solutions could be obtained after hot-pressing at 1900°C. Similarly, the composition of $(Tb_{0.25}Er_{0.25}Yb_{0.25}Lu_{0.25})_2Si_2O_7$ exhibit dual-phase mixture containing 66.04 wt% β-phase and 33.96 wt% γ-phase solid solutions at 1550°C, which turn into 50.94 wt% β-phase and 49.06 wt% γ-phase at 1800°C (Supplementary Fig. 3b, and Supplementary Fig. 5c, d); and the compound is stabilized as single-phase γ-$(Tb_{0.25}Er_{0.25}Yb_{0.25}Lu_{0.25})_2Si_2O_7$ solid solutions at 1900°C. Therefore, the trend of phase formation and transformation for these two compositions could be described as the formation of β-$(RE^{I}_{0.25}RE^{II}_{0.25}RE^{III}_{0.25}RE^{IV}_{0.25})_2Si_2O_7$ solid solution followed by the occurrence of β→γ polymorphic transformation. In comparison with other compounds showing similar route of phase formation and transformation (such as $(Gd_{0.25}Tm_{0.25}Yb_{0.25}Lu_{0.25})_2Si_2O_7$ shown in Supplementary Fig. 2c), wherein single-phase γ-type $(RE^{I}_{0.25}RE^{II}_{0.25}RE^{III}_{0.25}RE^{IV}_{0.25})_2Si_2O_7$ solid solution could be achieved at relative lower temperature (~1800°C); the higher temperature (~1900°C) needed to obtain single-phase γ-$(Y_{0.25}Ho_{0.25}Tm_{0.25}Lu_{0.25})_2Si_2O_7$ and γ-$(Tb_{0.25}Er_{0.25}Yb_{0.25}Lu_{0.25})_2Si_2O_7$ could be attributed to the sluggish β→γ polymorphic transformation for these two compositions. Similar results have been observed in $(Yb_{0.5}Ho_{0.5})_2Si_2O_7$ ($\bar{r}$=0.885 Å and σ_r=0.017, also lie in the vicinity of the β-γ boundary) [7], wherein the synthesized sample exhibits dual-phase mixture of β-$(Yb_{0.5}Ho_{0.5})_2Si_2O_7$ and γ-$(Yb_{0.5}Ho_{0.5})_2Si_2O_7$ solid solutions. Here we note that, understanding the mechanisms of the sluggish β→γ polymorphic transformation near

the β-γ boundary, which probably involves complicated thermodynamic factors beyond the descriptive power of the $r(RE^{3+})$ related parameters, is a potentially important topic for future researches, but is beyond the scope of the present work.

Supplementary Note 4: The configurational entropy of mixing for ideal solid solutions

For an ideal solid solution system, the configurational entropy of mixing (S_{config}) could be formulated as proportional to the natural logarithm of the number of possible ways of occupation on individual atomic sites (the microstates), under the assumption that each microstate has equal probability. For the multicomponent $RE_2Si_2O_7$, the ideal S_{config} could be estimated from: [10]

$$S_{\text{config}}^{\infty} = -R\left[2\sum_{i=1}^{N^{\text{RE}}} x_i \ln(x_i) + 2\sum_{j=1}^{N^{\text{Si}}} x_j \ln(x_j) + 7\sum_{k=1}^{N^{\text{O}}} x_k \ln(x_k)\right] \quad (1)$$

where R=8.31 J·mol^{-1}·K^{-1} is the idea gas constant; N^{RE}, N^{Si} and N^{O} are the number of components in the sublattice of RE, Si and O, and x_i, x_j and x_k are their molar fraction. The calculated $S_{\text{config}}^{\infty}$ of $(RE^{I}_{0.25}RE^{II}_{0.25}RE^{III}_{0.25}RE^{IV}_{0.25})_2Si_2O_7$ is 2.77R, equaling to 23.04 J·mol^{-1}·K^{-1}, wherein the contributions from Si sites and O sites are ideally zero. There are two tips that should be kept in mind. Firstly, the $S_{\text{config}}^{\infty}$ parameter should only be valid for ideal solid solutions, which is why we mark it with an "∞". It could be understood as the higher limit of S_{config} that correspond to the perfectly disordered crystalline with infinitely large lattice and totally random occupations on the doping sites. Secondly, the $S_{\text{config}}^{\infty}$ parameter should only be dependent on the specific formula of the compound, the number of principal components on the doping sites as well as the molar fractions; whereas irrelevant to the combination of elemental species

and the polymorphic types.

Supplementary Note 5: The formation energy of $(RE^{I}_{0.25}RE^{II}_{0.25}RE^{III}_{0.25}RE^{IV}_{0.25})_2Si_2O_7$ compounds

To illustrate the enthalpic stability of β and γ phases, we calculated the formation energy for all the metastable configurations in each ensemble, wherein the RE_2O_3 and SiO_2 oxides as taken as reference states. As shown in the Supplementary Fig. 9, the formation energies (E_i) for all the metastable configurations in the β-type and γ-type $(Dy_{0.25}Tm_{0.25}Yb_{0.25}Lu_{0.25})_2Si_2O_7$, $(Dy_{0.25}Ho_{0.25}Yb_{0.25}Lu_{0.25})_2Si_2O_7$ and $(Gd_{0.25}Ho_{0.25}Yb_{0.25}Lu_{0.25})_2Si_2O_7$ ensemble are below zero, indicating the enthapic stability of these configurations. The lower E_i values for β-type structure than the γ-type structure demonstrates the higher enthalpic stability for the β-phases. This agrees with the normal trend of DFT calculations, that the low-temperature phase (β) observed from the experiments will be predicted to show lower energy than the high-temperature phase (γ). By contrast, the β-type and γ-type $(La_{0.25}Ce_{0.25}Yb_{0.25}Lu_{0.25})_2Si_2O_7$ ensemble show quite wide energy spread. Interestingly, many metastable configurations have $E_i<0$, which indicates that these configurations, individually by themselves, might be formed on the enthalpic basis; but there are also many configurations showing enthalpic instability ($E_i>0$). In such cases, the formation energy of any randomly chosen configuration is biased in addressing the formation of the multicomponent system; rather, it is the ensemble behaviors averaged over multiple metastable configurations that matters.

Supplementary Note 6: Stochastic generation of the configuration ensemble

The available configurations for the multi-RE-principal-component $RE_2Si_2O_7$ solid solution are generated by constructing periodic supercells with ordered structure to imitate the randomness for disordered crystallines, and sampling on different shape of the supercells as well as different occupation of the RE cation sites. This could be done by employing the special quasirandom structures (SQS) generation code implemented in the Alloy Theoretic Automated Toolkit (ATAT) package [11]. In practice, the size of supercells should be carefully chosen to effectively represent the randomized lattice occupation without triggering unbearable computational load. In the case of β- and γ-type $(RE^{I}_{0.25}RE^{II}_{0.25}RE^{III}_{0.25}RE^{IV}_{0.25})_2Si_2O_7$ materials investigated here, supercells with 88 lattice sites are used, corresponding to four times of the minimum cell size necessary to reproduce the required stoichiometry. The anion sites of supercells are occupied with 56 O atoms (occupancy probability of 1.00); whereas the cation sites are occupied by 16 RE atoms (4 RE^{I}, 4 RE^{II}, 4 RE^{III} and 4 RE^{IV}, with occupancy probability of 0.25 for each type), as well as 16 Si atoms (occupancy probability of 1.00). The algorithm initially enumerates 93 and 29 superlattices for the β- and γ-type lattices, respectively. For each superlattice, several possible RE cationic occupations, out of totally $C_{16}^{4}C_{12}^{4}C_{8}^{4}C_{4}^{4}$ combinations for the assignment of $RE^{I}/RE^{II}/RE^{III}/RE^{IV}$ atoms onto the RE cation sites, are randomly chosen to ensure that the configuration space is extensively searched and not biased by a pre-specified atomic occupation. Subsequently, all the generated configurations are collected and checked for uniqueness, wherein the configurations showing duplicated total energy

or atomic occupation are eliminated from the ensemble. In this way, it is guaranteed that all the constructed metastable configurations are different from each other and thus potentially explores the far extent of the energy spread of the complete ensemble. Alternatively, some other more complex sampling procedures for the generation of metastable configurations, such as using the genetic algorithm, or including some clustered atomic configurations etc., could be employed to promote extensive representation of the energy space of the ensemble. Nevertheless, such procedures are too computationally expensive for the $(RE^{I}_{0.25}RE^{II}_{0.25}RE^{III}_{0.25}RE^{IV}_{0.25})_2Si_2O_7$ materials, which have large primitive unit cell and low crystal symmetry.

In addition, we performed convergence test on the energy spread of metastable configurations, as well as the enthalpy, free energy, and configurational entropy of mixing for the ensemble, by changing the total number of configurations contained in the ensemble. Supplementary Fig. 10 presents the results of convergence test on β-type and γ-type $(Dy_{0.25}Ho_{0.25}Yb_{0.25}Lu_{0.25})_2Si_2O_7$ configuration ensembles. As is shown in Supplementary Fig. 10a-d, the energy spread of the metastable β-$(Dy_{0.25}Ho_{0.25}Yb_{0.25}Lu_{0.25})_2Si_2O_7$ configurations show similar features as the size of ensemble expands. The calculated enthalpy, free energy, and configurational entropy of mixing show good convergence for the ensembles containing several hundred of metastable configurations; whereas too small size of ensemble (such as N<100, N denotes the number of metastable configurations contained in the ensemble) might results in the enthalpy of mixing to deviate away from the others. The convergence test for the γ-$(Dy_{0.25}Ho_{0.25}Yb_{0.25}Lu_{0.25})_2Si_2O_7$ configuration ensemble shows similar

results (Supplementary Fig. 10e-h). What worthy of noticing is that, despite that relatively smaller size of ensemble (such as N=186 for the β-type ensemble and N=116 for γ-type ensemble) could properly interpret the thermodynamic state functions of the $(RE^{I}_{0.25}RE^{II}_{0.25}RE^{III}_{0.25}RE^{IV}_{0.25})_2Si_2O_7$ ensembles; it might cause rather coarse Gaussian-fit of the energy distribution spectrum, and accordingly, inaccurate extraction of FWHM parameters as discussed in the main manuscript. Therefore, in this study, the configuration ensembles are constructed to include 558 unique configurations for the β-type $(RE^{I}_{0.25}RE^{II}_{0.25}RE^{III}_{0.25}RE^{IV}_{0.25})_2Si_2O_7$, and 319 for the γ-type materials, which is expected to give reliable prediction on the energy distribution spectrum and the thermodynamic state functions, while maintaining acceptable computational load. In fact, ensembles in similar sizes (containing metastable configurations in the order of ~10^2) have been reported to give reliable simulations on the thermodynamic and mechanical properties of other multicomponent systems, such as high-entropy metal oxides and metal carbides.[12,13]

Supplementary Note 7: Convergence test on the energy cut-off for DFT calculations

We performed convergence test on the energy spread, i.e., the single-point energies (E) of the testing configurations minus their average values ($\bar{E}$), for the material system with respect to increased energy cut-offs. Herein, the β-$(Dy_{0.25}Tm_{0.25}Yb_{0.25}Lu_{0.25})_2Si_2O_7$ and the γ-$(Gd_{0.25}Ho_{0.25}Yb_{0.25}Lu_{0.25})_2Si_2O_7$ systems are taken for examples, which represent the different polymorphic types as well as the situations of narrower and wider energy distributions (Fig. 5a in the main manuscript)

discussed in this study. For each system, sixteen configurations are chosen as representatives; and, their energies are examined to span over the entire energy range of each ensemble, so as to ensure the reliability of the convergence test. As shown in the Supplementary Fig. 11a, the $E-\bar{E}$ values of the sixteen representative configurations remain quite close as the energy cut-off increases from 500 eV to 800 eV; whereas the data for the energy cut-off of 400 eV deviates obviously from the others. Meanwhile, the root-mean-square of the energies for the sixteen configurations converge well when the energy cut-off is higher than 500 eV. Similar results could be found for the γ-$(Gd_{0.25}Ho_{0.25}Yb_{0.25}Lu_{0.25})_2Si_2O_7$ system (Supplementary Fig. 11b). These results demonstrate that the chosen energy cut-off (600 eV, 1.5 times of the maximum ENMAX values from the POTCAR file) in DFT calculations could give reliable prediction on the energy distribution of the configuration ensembles.

Supplementary Figures

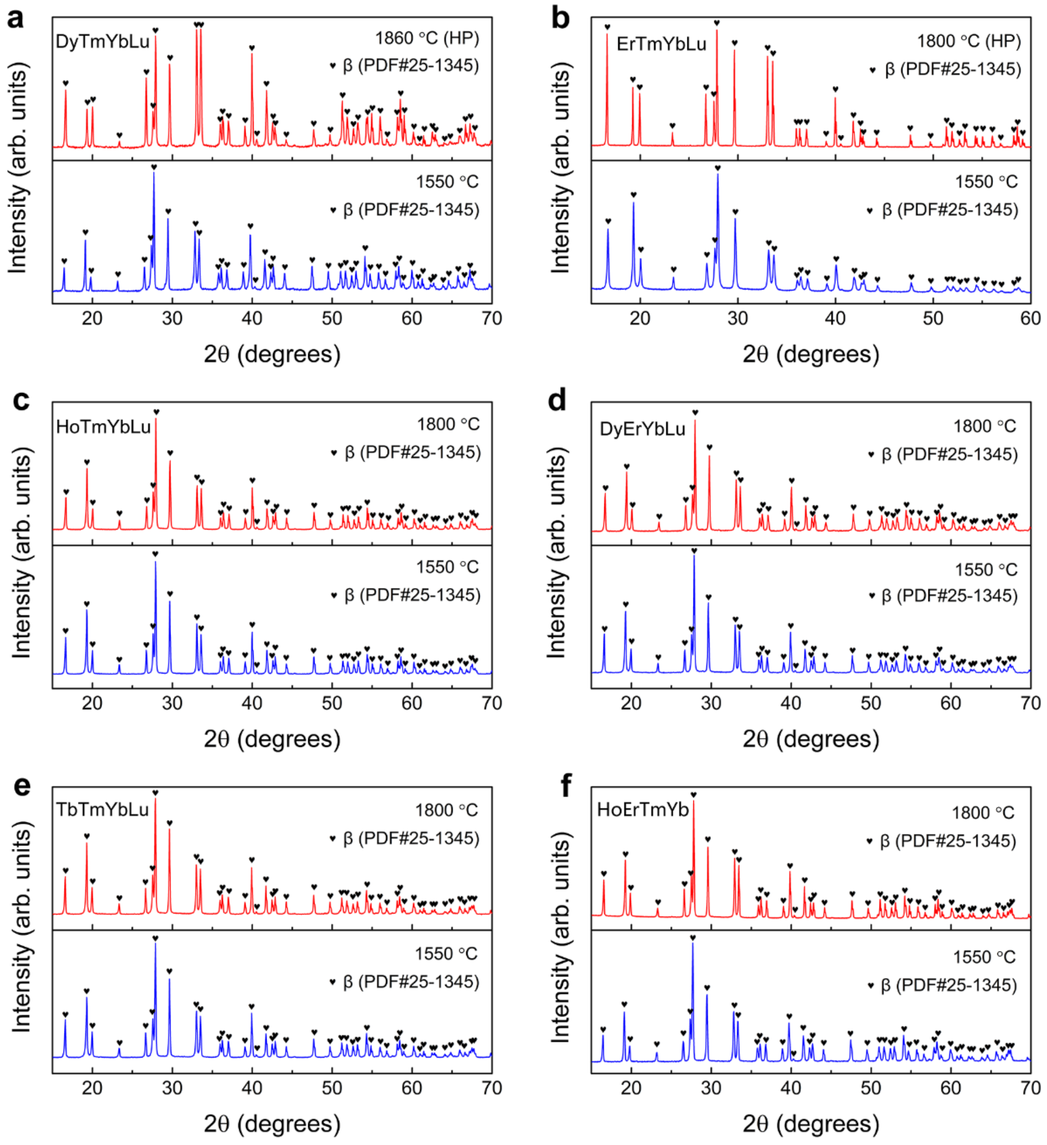

Supplementary Figure 1. XRD patterns of the synthesized $(RE^{I}_{0.25}RE^{II}_{0.25}RE^{III}_{0.25}RE^{IV}_{0.25})_2Si_2O_7$ samples belonging to the category I. Results for the composition of **a** $(Dy_{0.25}Tm_{0.25}Yb_{0.25}Lu_{0.25})_2Si_2O_7$, **b** $(Er_{0.25}Tm_{0.25}Yb_{0.25}Lu_{0.25})_2Si_2O_7$, **c** $(Ho_{0.25}Tm_{0.25}Yb_{0.25}Lu_{0.25})_2Si_2O_7$, **d** $(Dy_{0.25}Er_{0.25}Yb_{0.25}Lu_{0.25})_2Si_2O_7$, **e** $(Tb_{0.25}Tm_{0.25}Yb_{0.25}Lu_{0.25})_2Si_2O_7$, and **f** $(Ho_{0.25}Er_{0.25}Tm_{0.25}Yb_{0.25})_2Si_2O_7$ samples prepared at 1550°C and 1800°C/1860°C. “HP” denotes the XRD patterns for bulk samples fabricated via hot-pressing sintering method.

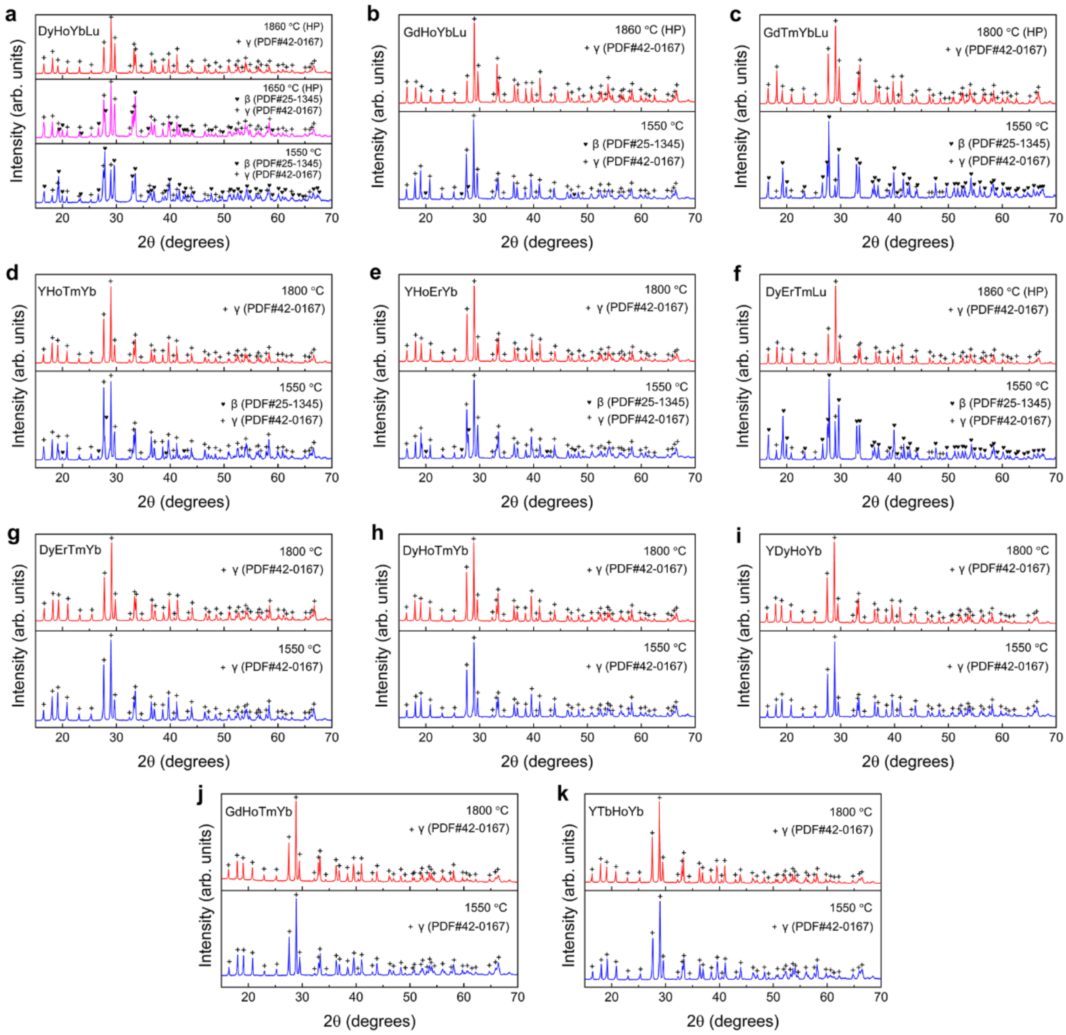

Supplementary Figure 2. XRD patterns of the synthesized $(RE^{I}_{0.25}RE^{II}_{0.25}RE^{III}_{0.25}RE^{IV}_{0.25})_2Si_2O_7$ samples belonging to the category II. Results for the composition of **a** $(Dy_{0.25}Ho_{0.25}Yb_{0.25}Lu_{0.25})_2Si_2O_7$, **b** $(Gd_{0.25}Ho_{0.25}Yb_{0.25}Lu_{0.25})_2Si_2O_7$, **c** $(Gd_{0.25}Tm_{0.25}Yb_{0.25}Lu_{0.25})_2Si_2O_7$, **d** $(Y_{0.25}Ho_{0.25}Tm_{0.25}Yb_{0.25})_2Si_2O_7$, **e** $(Y_{0.25}Ho_{0.25}Er_{0.25}Yb_{0.25})_2Si_2O_7$, and **f** $(Dy_{0.25}Er_{0.25}Tm_{0.25}Lu_{0.25})_2Si_2O_7$, **g** $(Dy_{0.25}Er_{0.25}Tm_{0.25}Yb_{0.25})_2Si_2O_7$, **h** $(Dy_{0.25}Ho_{0.25}Tm_{0.25}Yb_{0.25})_2Si_2O_7$, **i** $(Y_{0.25}Dy_{0.25}Ho_{0.25}Yb_{0.25})_2Si_2O_7$, **j** $(Gd_{0.25}Ho_{0.25}Tm_{0.25}Yb_{0.25})_2Si_2O_7$, and **k** $(Y_{0.25}Tb_{0.25}Ho_{0.25}Yb_{0.25})_2Si_2O_7$ samples prepared at 1550°C and 1800°C/1860°C. "HP" denotes the XRD patterns for bulk samples fabricated via hot-pressing sintering method.

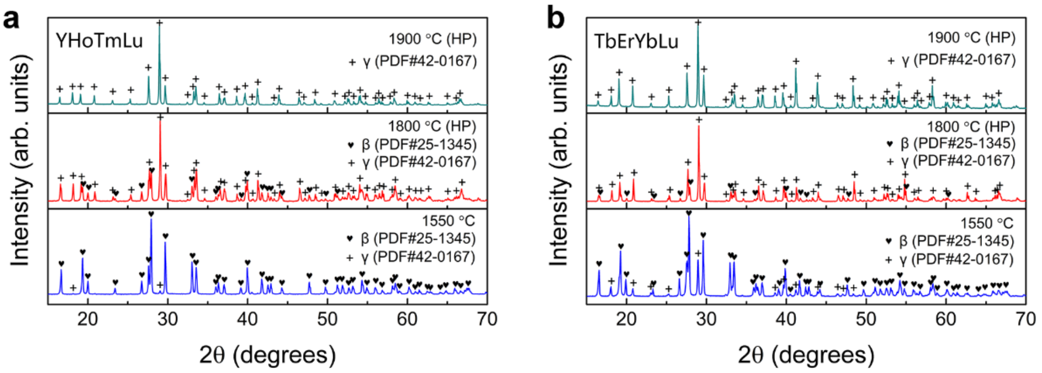

Supplementary Figure 3. XRD patterns of the synthesized $(RE^{I}_{0.25}RE^{II}_{0.25}RE^{III}_{0.25}RE^{IV}_{0.25})_2Si_2O_7$ samples belonging to the category II and showing sluggish β→γ polymorphic transformation. Results for the composition of **a** $(Y_{0.25}Ho_{0.25}Tm_{0.25}Lu_{0.25})_2Si_2O_7$; and **b** $(Tb_{0.25}Er_{0.25}Yb_{0.25}Lu_{0.25})_2Si_2O_7$ samples prepared at 1550°C, 1800°C and 1900°C. "HP" denotes the XRD patterns for bulk samples fabricated via hot-pressing sintering method.

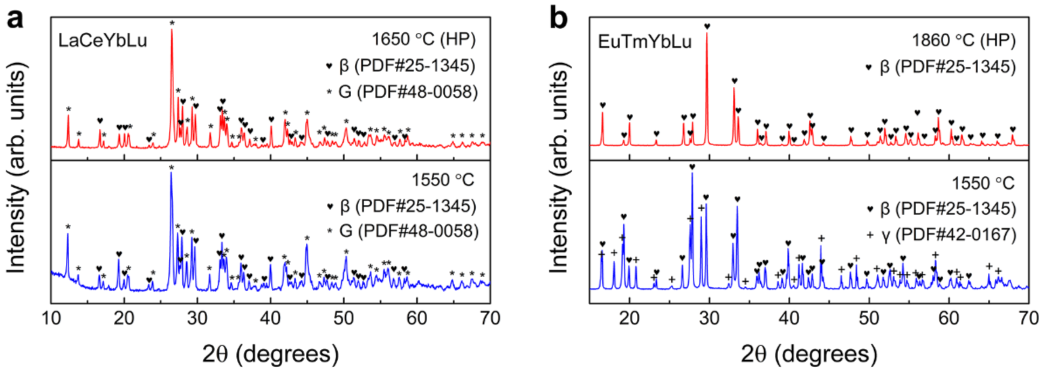

Supplementary Figure 4. XRD patterns of the synthesized $(RE^{I}_{x1}RE^{II}_{x2}RE^{III}_{x3}RE^{IV}_{x4})_2Si_2O_7$ (x1+x2+x3+x4=1) samples belonging to the category III. Results for the composition of **a** $(La_{x1}Ce_{x2}Yb_{x3}Lu_{x4})_2Si_2O_7$ samples prepared at 1550°C and 1650°C, and **b** $(Eu_{x1}Tm_{x1}Yb_{x3}Lu_{x4})_2Si_2O_7$ samples prepared at 1550°C and 1860°C. “HP” denotes the XRD patterns for bulk samples fabricated via hot-pressing sintering method.

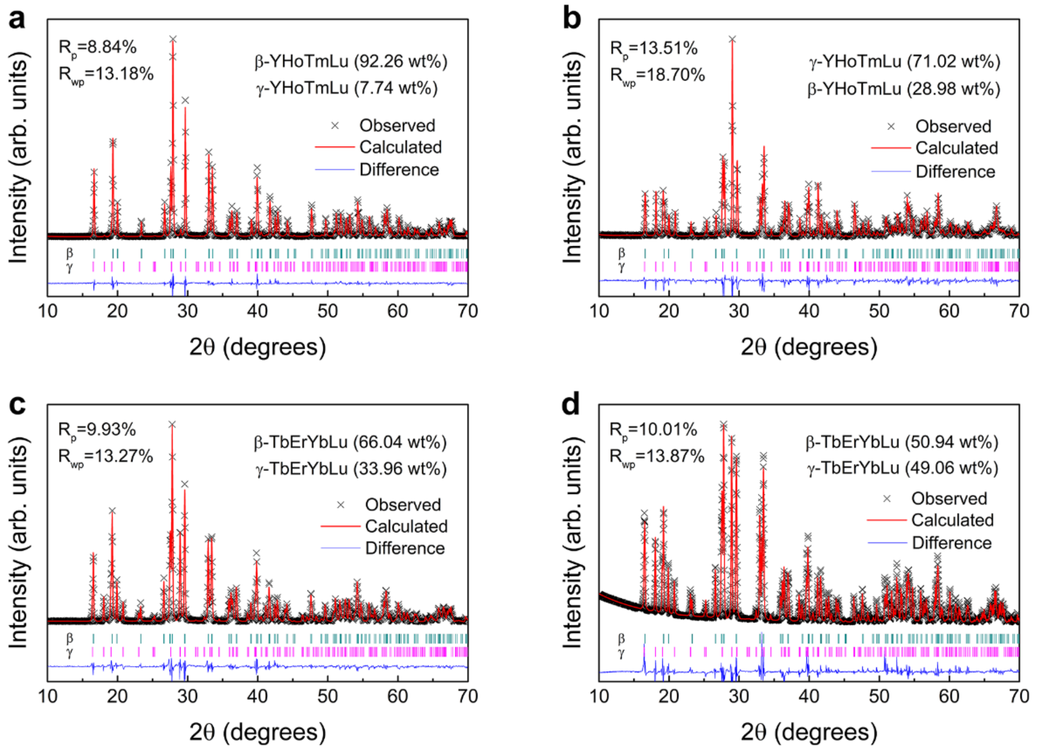

Supplementary Figure 5. Rietveld refinement of room-temperature XRD patterns for the studied materials. **a** Results for the as-synthesized powders of $(Y_{0.25}Ho_{0.25}Tm_{0.25}Lu_{0.25})_2Si_2O_7$ prepared at 1550°C. **b** Results for the bulk sample of $(Y_{0.25}Ho_{0.25}Tm_{0.25}Lu_{0.25})_2Si_2O_7$ fabricated via hot-pressing sintering method at 1800°C. **c** Results for the as-synthesized powders of $(Tb_{0.25}Er_{0.25}Yb_{0.25}Lu_{0.25})_2Si_2O_7$ prepared at 1550°C. **d** Results for the bulk sample of $(Tb_{0.25}Er_{0.25}Yb_{0.25}Lu_{0.25})_2Si_2O_7$ fabricated via hot-pressing sintering method at 1800°C. The reliability factors (R_p and R_{wp}) are also presented in the figures. The short vertical lines in dark cyan and magenta color at the bottom of each panel denote the positions of XRD peaks of β-$RE_2Si_2O_7$ and γ-$RE_2Si_2O_7$, respectively.

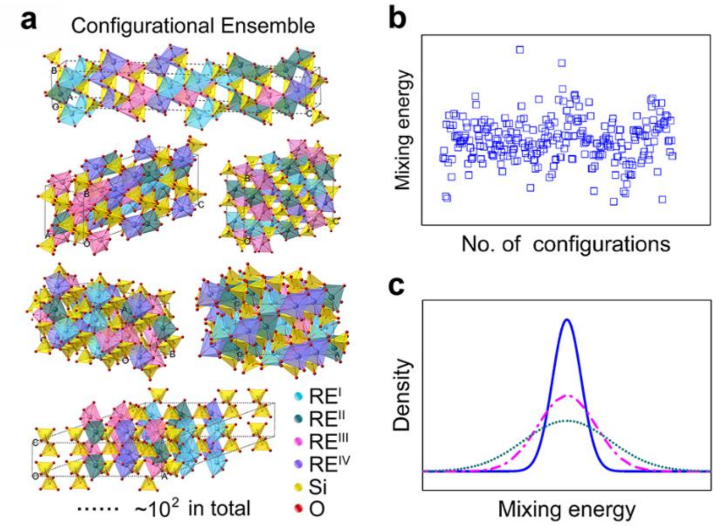

Supplementary Figure 6. The framework of the high-throughput calculations on the mixing energies. **a** Construction of the configuration ensemble for a given $(RE^{I}_{0.25}RE^{II}_{0.25}RE^{III}_{0.25}RE^{IV}_{0.25})_2Si_2O_7$ material, by sampling on different shape of the supercells and different occupation of the RE cation sites, and each ensemble contains several hundreds of stochastically generated configurations. **b** Schematic plot of the calculated mixing energy for all configurations in the ensemble. **c** Schematic plot of narrower and wider energy distribution, i.e., the energy distribution curves are narrowed in the sequence of cyan dot line, pink dash dot line, and blue solid line.

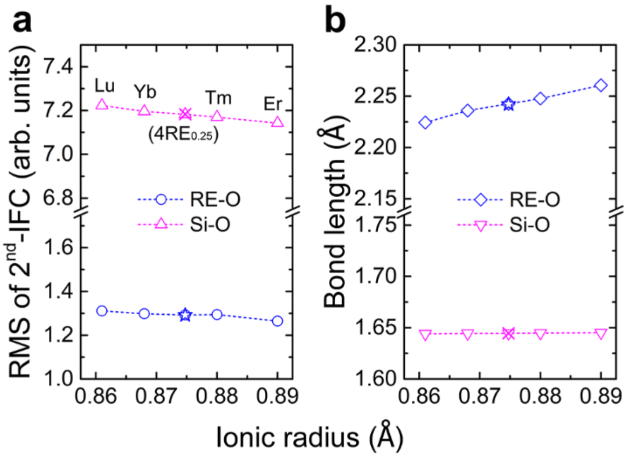

Supplementary Figure 7. The bonding characteristics of β-$RE_2Si_2O_7$ compounds. **a** The root-mean-square (RMS) of second-order interatomic force constant (IFC) for RE-O and Si-O bonds in the β-$(Er_{0.25}Tm_{0.25}Yb_{0.25}Lu_{0.25})_2Si_2O_7$ (herein marked as ($4RE_{0.25}$)) and single-RE-principal-component β-$RE_2Si_2O_7$ (RE = Er, Tm, Yb and Lu) lattices, plotted as a function of individual or average RE^{3+} radius. The IFC values are obtained from DFT-based phonon calculations. **b** The bond length of RE-O and Si-O bonds in these compounds. The pink cross and blue star represent the average values for Si-O and RE-O bonds, respectively, averaged over the four single-RE-principal-component β-$RE_2Si_2O_7$ (RE = Er, Tm, Yb and Lu) materials.

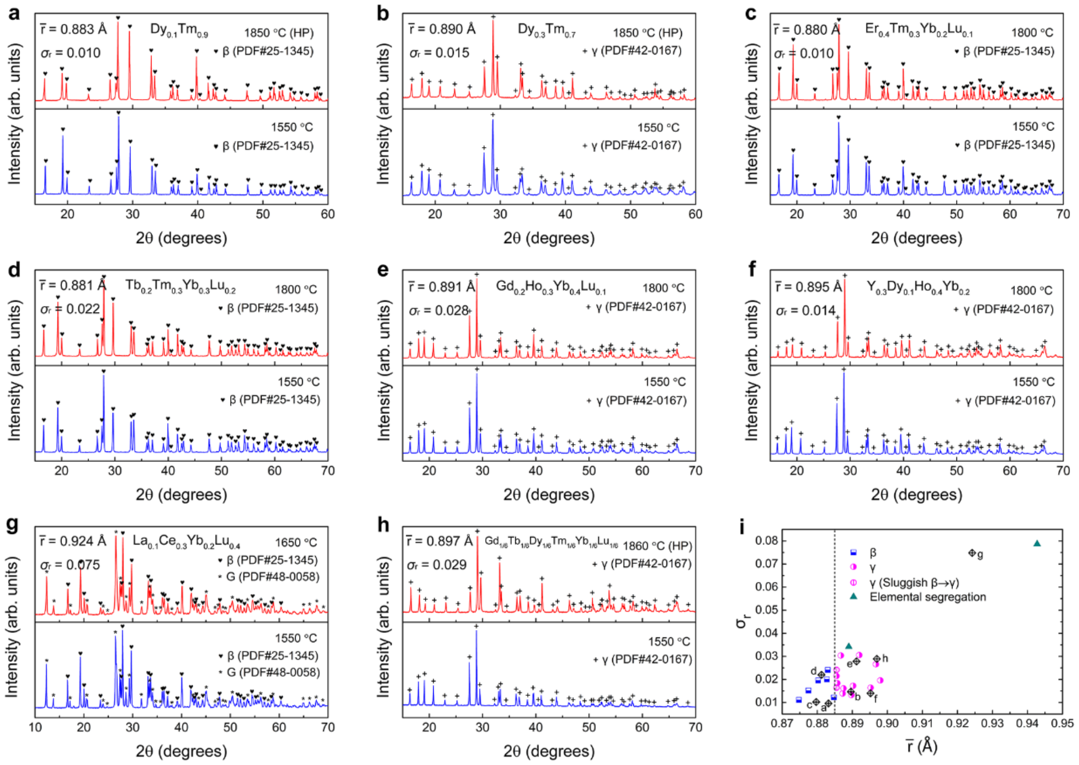

Supplementary Figure 8. XRD patterns of the synthesized equimolar and non-equimolar $(n\text{RE}_{xi})_2\text{Si}_2\text{O}_7$ (n=2, 4 and 6; $0 \le x_i \le 1$) compounds. Results for the composition of **a** $(\text{Dy}_{0.1}\text{Tm}_{0.9})_2\text{Si}_2\text{O}_7$ and **b** $(\text{Dy}_{0.3}\text{Tm}_{0.7})_2\text{Si}_2\text{O}_7$ samples prepared at 1550°C and 1850°C; **c** $(\text{Er}_{0.4}\text{Tm}_{0.3}\text{Yb}_{0.2}\text{Lu}_{0.1})_2\text{Si}_2\text{O}_7$, **d** $(\text{Tb}_{0.2}\text{Tm}_{0.3}\text{Yb}_{0.3}\text{Lu}_{0.2})_2\text{Si}_2\text{O}_7$, **e** $(\text{Gd}_{0.2}\text{Ho}_{0.3}\text{Yb}_{0.4}\text{Lu}_{0.1})_2\text{Si}_2\text{O}_7$, and **f** $(\text{Y}_{0.3}\text{Dy}_{0.1}\text{Ho}_{0.4}\text{Yb}_{0.2})_2\text{Si}_2\text{O}_7$ samples prepared at 1550°C and 1800°C; **g** $(\text{La}_{0.1}\text{Ce}_{0.3}\text{Yb}_{0.2}\text{Lu}_{0.4})_2\text{Si}_2\text{O}_7$ samples prepared at 1550°C and 1650°C; and **h** $(\text{Gd}_{1/6}\text{Tb}_{1/6}\text{Dy}_{1/6}\text{Tm}_{1/6}\text{Yb}_{1/6}\text{Lu}_{1/6})_2\text{Si}_2\text{O}_7$ samples prepared at 1550°C and 1860°C. "HP" denotes the XRD patterns for bulk samples fabricated via hot-pressing sintering method. **i** The average RE^{3+} radius ($\bar{r}$) and the deviations (σ_r) for all the equimolar and non-equimolar $(n\text{RE}_{xi})_2\text{Si}_2\text{O}_7$ compounds discussed in this study. Herein, the diamond symbols (with indices a~h) correspond to the above-mentioned compositions; whereas the other datapoints are duplicated from Fig. 4a in the main manuscript.

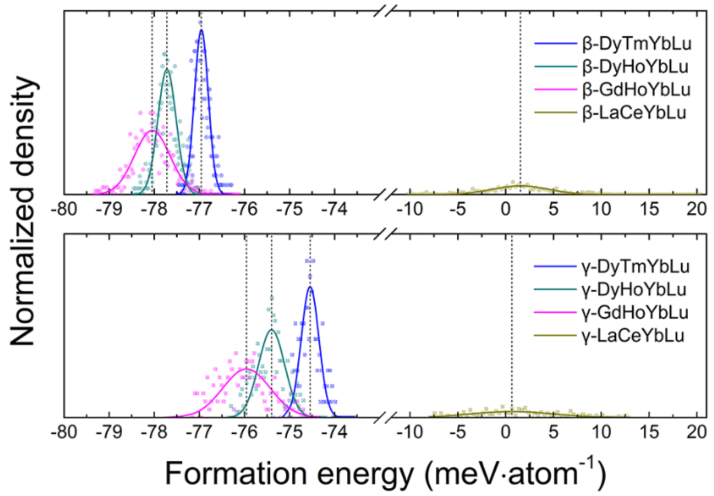

Supplementary Figure 9. The distribution spectrum of formation energy and the Gaussian fitting for all the metastable configurations in the studied ensembles. The scatters are the normalized density for formation energy (E_i); and the solid lines are the Gaussian fitting of the E_i distribution spectrum.

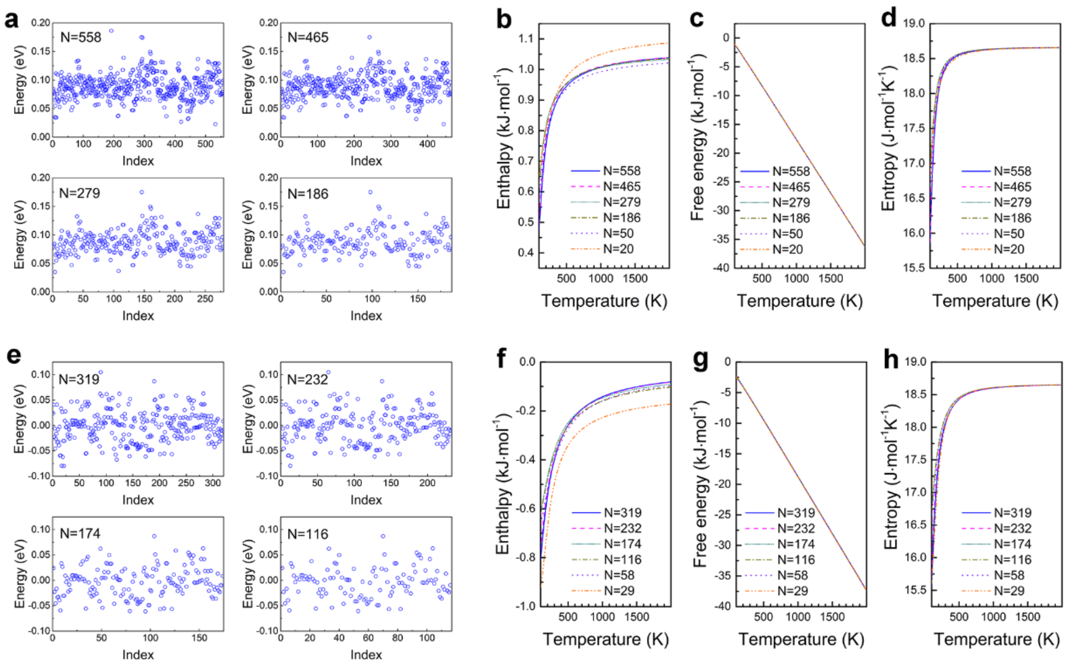

Supplementary Figure 10. Results of convergence test on the size of β-type and γ-type $(Dy_{0.25}Ho_{0.25}Yb_{0.25}Lu_{0.25})_2Si_2O_7$ configuration ensembles. **a** The spread of mixing energy of multiple configurations, as varied with the size of β-$(Dy_{0.25}Ho_{0.25}Yb_{0.25}Lu_{0.25})_2Si_2O_7$ ensemble. The **b** enthalpy, **c** free energy, and **d** configurational entropy of mixing (per formula) calculated using different size of β-$(Dy_{0.25}Ho_{0.25}Yb_{0.25}Lu_{0.25})_2Si_2O_7$ ensemble. **e**, **f**, **g** and **h** are the results for the γ-$(Dy_{0.25}Ho_{0.25}Yb_{0.25}Lu_{0.25})_2Si_2O_7$ ensemble. Herein, the parameter *N* denotes the number of metastable configurations contained in the ensemble.

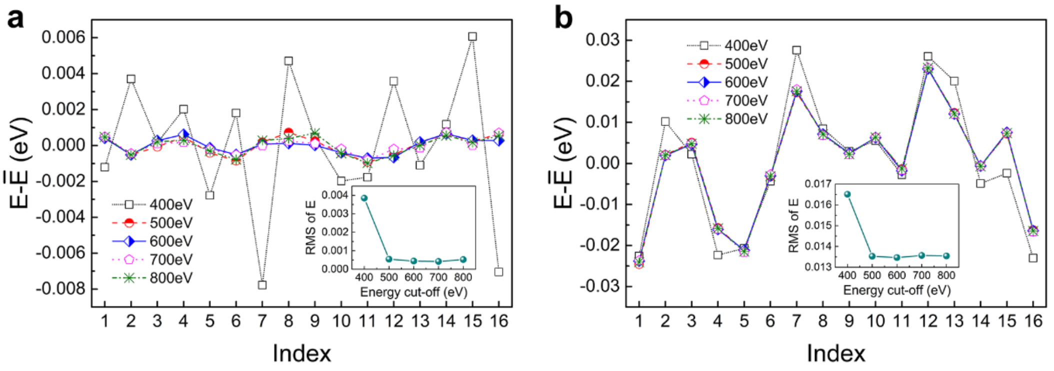

Supplementary Figure 11. Results of the convergence test on the energy cut-off in DFT calculations. The difference between the single-point energies (*E*) of the representative configurations and their average values ($\bar{E}$) for **a** β-$(Dy_{0.25}Tm_{0.25}Yb_{0.25}Lu_{0.25})_2Si_2O_7$, and **b** γ-$(Gd_{0.25}Ho_{0.25}Yb_{0.25}Lu_{0.25})_2Si_2O_7$ structures, calculated with the energy cut-off set as 400, 500, 600, 700 and 800 eV. Insets: The root-mean-square (RMS) of energies for the sixteen configurations plotted as a function of the energy cut-off.

Supplementary Tables

Supplementary Table 1. The atomic ratios of RE elements (in at. %) in different samples.

Samples	La	Ce	Dy	Ho	Yb	Lu
β-$(Dy_{x1}Ho_{x2}Yb_{x3}Lu_{x4})_2Si_2O_7$	/	/	24.22	21.49	27.74	26.55
γ-$(Dy_{x1}Ho_{x2}Yb_{x3}Lu_{x4})_2Si_2O_7$	/	/	24.70	23.01	25.70	26.59
β-$(La_{x1}Ce_{x2}Yb_{x3}Lu_{x4})_2Si_2O_7$	1.53	3.05	/	/	44.61	50.81
G-$(La_{x1}Ce_{x2}Yb_{x3}Lu_{x4})_2Si_2O_7$	32.50	32.24	/	/	18.20	17.06

Note: The results are analyzed based on the TEM-EDS mapping, shown in the Fig. 3d, h, l, and p in the main manuscript.

Supplementary References

1. Bondar, I. A. Bondar-Rare-earth silicates. *Ceram. Int.* **8**, 83-89 (1982).
2. Ohashi, H., Alba, M. D., Becerro, A. I., Chain, P. & Escudero, A. Structural study of the $Lu_2Si_2O_7$-$Sc_2Si_2O_7$ system. *J. Phys. Chem. Solids* **68**, 464-469 (2007).
3. Fernández-Carrión, A. J., Alba, M. D., Escudero, A. & Becerro, A. I. Solid solubility of $Yb_2Si_2O_7$ in β-, γ- and δ-$Y_2Si_2O_7$. *J. Solid State Chem.* **184**, 1882-1889 (2011).
4. Escudero, A., Alba, M. D. & Becerro, A. I. Polymorphism in the $Sc_2Si_2O_7$-$Y_2Si_2O_7$ system. *J. Solid State Chem.* **180**, 1436-1445 (2007).
5. Becerro, A. I. & Escudero, A. Polymorphism in the $Lu_{2-x}Y_xSi_2O_7$ system at high temperatures. *J. Eur. Ceram. Soc.* **26**, 2293-2299 (2006).
6. Becerro, A. I. & Escudero, A. Phase transitions in Lu-doped $Y_2Si_2O_7$ at high temperatures. *Chem. Mater.* **17**, 112-117 (2005).
7. Lv, X., Cui, J., Zhang, J. & Wang, J. Phase composition and property evolution of $(Yb_{1-x}Ho_x)_2Si_2O_7$ solid solution as environmental/thermal barrier coating candidates. *J. Eur. Ceram. Soc.* **42**, 4377-4387 (2022).
8. Dong, Y. et al. High-entropy environmental barrier coating for the ceramic matrix composites. *J. Eur. Ceram. Soc.* **39**, 2574-2579 (2019).
9. Sun, L. et al. A multicomponent gamma-type $(Gd_{1/6}Tb_{1/6}Dy_{1/6}Tm_{1/6}Yb_{1/6}Lu_{1/6})_2Si_2O_7$ disilicate with outstanding thermal stability. *Mater. Res. Lett.* **8**, 424-430 (2020).
10. Sarkar, A., Breitung, B. & Hahn, H. High entropy oxides: The role of entropy, enthalpy and synergy. *Scripta Mater.* **187**, 43-48 (2020).
11. van de Walle, A. et al. Efficient stochastic generation of special quasirandom structures. *Calphad* **42**, 13-18 (2013).
12. Anand, G., Wynn, A. P., Handley, C. M. & Freeman, C. L. Phase stability and distortion in high-entropy oxides. *Acta Mater.* **146**, 119-125 (2018).
13. Sarker, P. et al. High-entropy high-hardness metal carbides discovered by entropy descriptors. *Nat. Commun.* **9**, 4980 (2018).
